# Regulation of Mutant Huntingtin Mitochondrial Toxicity by Phosphomimetic Mutations within Its N-Terminal Region

**DOI:** 10.1523/JNEUROSCI.1254-24.2024

**Published:** 2025-01-08

**Authors:** Svitlana Yablonska, Colleen E. Strohlein, Sergei V. Baranov, Stacy M. Yeh, Aashka Patel, Tanisha Singh, Abhishek Jauhari, JinHo Kim, Nicolas K. Khattar, Fang Li, Xiaomin Wang, Yue-Fang Chang, C. Y. Daniel Lee, X. William Yang, Diane L. Carlisle, Robert M. Friedlander

**Affiliations:** ^1^Neuroapoptosis Laboratory, Department of Neurological Surgery, University of Pittsburgh School of Medicine, Pittsburgh, Pennsylvania 15213; ^2^ Center for Neurobehavioral Genetics, Semel Institute for Neuroscience & Human Behavior at UCLA, Los Angeles, California 90095; ^3^Department of Psychiatry & Biobehavioral Sciences, David Geffen School of Medicine at UCLA, Los Angeles, California 90095

**Keywords:** mitochondria, mutant huntingtin, phosphomimetic mutation

## Abstract

Huntington's disease (HD), a neurodegenerative disease, affects approximately 30,000 people in the United States, with 200,000 more at risk. Mitochondrial dysfunction caused by mutant huntingtin (mHTT) drives early HD pathophysiology. mHTT binds the translocase of the mitochondrial inner membrane (TIM23) complex, inhibiting mitochondrial protein import and altering the mitochondrial proteome. The 17 aa HTT N-terminal sequence (N17) acts as a regulatory domain in HD pathogenesis; phosphomimetic modification of serines 13 and 16 of the N17 domain impacts subcellular localization and degradation and ameliorates toxicity in mouse and cell models of HD. Using cellular and mouse (either sex) HD models, we investigated the mechanisms by which HTT phosphorylation affects intracellular localization. We demonstrate that introducing phosphomimetic mutations within the mHTT fragment N17 domain decreased TIM23 binding affinity and reduced inhibition of mHTT-mediated mitochondrial protein import. BACHD-SD mice expressing full-length mHTT harboring the same two N17 phosphomimetic mutations have an ameliorated HD-like phenotype as compared with mice expressing mHTT. Consistent with reduced toxicity in vivo, we found that the amount of full-length mHTT in the brain mitochondria of BACHD-SD transgenic mice is less when the mHTT has two phosphomimetic mutations. To complement the relevance of the phosphomimetic HTT findings, endogenous N17 phospho-mHTT is less likely to translocate to the mitochondria compared with nonphosphorylated mHTT. We demonstrate that phosphorylation of mHTT at serines 13 and 16 is critical for negatively regulating mHTT mitochondrial targeting and that reducing mHTT mitochondrial localization and binding to TIM23 results in amelioration of mHTT-induced mitochondrial and neuronal toxicity.

## Significance Statement

We establish the first 17 aa of mutant huntingtin (mHTT) as a mitochondrial targeting sequence driving mHTT accumulation in mitochondria and show that N-terminal phosphomimetic modifications reduce mitochondrial accumulation and toxicity. Utilizing super-resolution live imaging and expansion microscopy of isolated mitochondria and full-length mHTT immunoprecipitation from human cells, we demonstrate HTT intramitochondrial localization and interaction with the mitochondrial protein importing complex. mHTT N17 domain phosphomimetic mutations reduce binding affinity for TIM23 subunit, preventing mHTT mitochondrial accumulation. This lessens mHTT inhibitory effect on mitochondrial protein import. Our study elucidates the molecular mechanism responsible for BACHD-SD mice ameliorated phenotype and underscores a critical need to identify enzymes responsible for regulating HTT phosphorylation to exploit their potential as therapeutic Huntington’s disease targets.

## Introduction

Huntington's disease (HD) is an autosomal dominant, universally fatal hereditary neurodegenerative disorder characterized primarily by selective neuronal loss in the striatum and cortex caused by the mutant huntingtin (mHTT) protein. Symptoms of HD include involuntarily movement disorder and both cognitive and psychiatric disturbances. HTT is a 350 kD ubiquitously expressed protein containing a polyglutamine (polyQ) stretch after a 17-aa-long leader sequence at the N-terminal end. Elongation of HTT's polyQ region to over 35 repeats results in selective pathological cellular toxicity (Gusella et al., 1993). A crucial driver of HD pathophysiology is mitochondrial dysfunction (Choo et al., 2004; Orr et al., 2008; Yano et al., 2014; Guo et al., 2016; Yablonska et al., 2019) resulting from direct mHTT interactions in the mitochondria. Mitochondrial dysfunction has been demonstrated in cells and tissues expressing mHTT, such as inhibition of mitochondrial protein import and disbalance of the mitochondrial proteome (Yano et al., 2014; Yablonska et al., 2019), mitochondrial calcium dyshomeostasis (Choo et al., 2004; Milakovic et al., 2006; Giacomello et al., 2013), and defects in mitochondrial energy metabolism (Milakovic and Johnson, 2005; Seong et al., 2005). Given that mitochondria import over 99% of the proteins required for proper function (Sickmann et al., 2003; Taylor et al., 2003), regulation of this process is critical for normal mitochondrial function and cellular health. We previously demonstrated an early (presymptomatic) finding of synaptic mitochondrial inhibition of protein import resulting from a direct high-affinity interaction between mHTT and TIM23 (Yano et al., 2014; Yablonska et al., 2019). Furthermore, the first 17 aa of HTT are required for the interaction of HTT with TIM23 (Yano et al., 2014). Since HTT and mHTT reside in the mitochondria and mHTT is detrimental to mitochondrial function, it is critical to understand the mechanisms of HTT mitochondrial targeting to mitigate mHTT-mediated mitochondrial pathology.

Huntingtin is predominantly a cytoplasmic protein, with a nuclear targeting signal at the C terminus (Xia et al., 2003) and an N-terminal α-helical domain with membrane-binding properties (Atwal et al., 2007; Rockabrand et al., 2007; Pandey et al., 2018), that regulates association with mitochondria, endoplasmic reticulum, and Golgi apparatus (Atwal et al., 2007; Maiuri et al., 2013; Brandstaetter et al., 2014). Recent studies have suggested a regulatory role of the N-terminal domain in the pathogenesis of HD. The 17 aa N-terminal sequence (N17) of HTT impacts its subcellular localization (Atwal et al., 2007; Rockabrand et al., 2007; Maiuri et al., 2013) and degradation (Thompson et al., 2009). Double phosphorylation of serines 13 and 16 affects N17 conformation and modifies subcellular localization of wild-type HTT, directing it to the nucleus (Atwal et al., 2011). Additionally, a mouse model (Gu et al., 2009) expressing full-length mHTT was genetically modified to mimic phosphorylation of HTT within the first 17 aa (N17) by converting serines 13 and 16 to aspartic acid (S13D S16D; BACHD-SD). BACHD-SD mice did not exhibit symptoms of HD when compared with HD mice expressing mHTT. Therefore, posttranscriptional phosphorylation at residues 13 and 16 of the N17 domain reduces mHTT toxicity in murine and cellular models of HD (Gu et al., 2009; Thompson et al., 2009) although the molecular mechanism for this is not understood. Considering the role of mHTT in mitochondrial pathology, we hypothesized that phosphomimetic modifications to mHTT's N17 domain may affect its translocation and molecular interactions in mitochondria, reducing mHTT-mediated mitochondrial pathology.

In this study, we confirmed that the first 17 N-terminal amino acids are HTT's mitochondrial targeting signal. Phosphomimetic mutations at serines 13 and 16 (S13E S16E or S13D S16D) mimicking posttranslational modifications in HTT, which significantly ameliorate mHTT-mediated toxicity in vivo (Gu et al., 2009) and in vitro (Atwal et al., 2011), disrupt HTT's mitochondrial targeting, reduce the affinity of mHTT for TIM23, and ameliorate the inhibitory effects of mHTT on mitochondrial protein import and cell death. Brain mitochondria isolated from BACHD-SD mice carrying mHTT with two phosphomimetic mutations show lower content of mHTT S13ES16E compared with mHTT. In summation, we confirm that HTT is targeted to the mitochondria by the N17 domain, that this domain is responsible for the interaction between HTT and TIM23, and that N17 phosphomimetics decrease both HTT mitochondrial targeting and TIM23 binding affinity. These results suggest that modulation of N17 phosphorylation may modulate mHTT-induced toxicity.

## Materials and Methods

### Ethics approval and consent to participate

All mammalian experiments were performed in compliance with the US National Institute of Health Guide for the Care and Use of Laboratory Animals. Animal protocols used were approved by the Institutional Animal Care and Use Committee at the University of Pittsburgh.

### Plasmids and recombinant proteins

pGEX-4T3 plasmid vectors expressing GST-fused recombinant protein (HTTexon1-97Q and -HTTexon1-97Q-S13ES16E, TIM23, TIM50, TIM17A, TIM17B) were generated as described previously (Yano et al., 2014). Proteins were purified from transformed One Shot BL21 Star (DE3) cells (Life Technologies) detailed procedure outlined by Yano et al. (2014). GST-TIM23, GST-TIM50, GST-TIM17A, and GST-TIM17B were digested with thrombin protease (GE HealthCare) to remove GST proteins and concentrated using Amicon Ultra Centrifugal Filter 10 K (Millipore).

Plasmid vectors for confocal microscopy were generated based on peGFP-N1 (Clontech). Double-stranded oligonucleotides coding 17-aa-long N-terminal sequence of human HTT (N17) and identical sequence with two point-mutations replacing nucleotides encoding serines 13 and 16 to glutamic acids (N17-S13ES16E) were inserted into multiple cloning site (MCS) of peGFP-N1. Mito-eGFP construct was generated by cloning the 25-aa-long mitochondrial targeting sequence of cytochrome *c* oxidase subunit 8A into MCS of peGFP-N1.

The overexpression of phosphomimetics and nonmutated counterparts of wild-type and mutant 171-aa-long HTT fragments in HEK293t was fulfilled by transfecting the cells with pcDNA3.1+ vectors which encoded HTT171-Q17-FLAG, HTT171-Q17-S13ES16E-FLAG, HTT171-Q68-FLAG, and HTT171-Q68-S13ES16E-FLAG. FLAG-tag encoding sequence (DYKDDDDK) was fused to the C terminus of the HTT171 product.

### Cell culture

ST-Hdh-Q7/Q7 cells were cultured in DMEM media supplemented with 5% FBS and 1% sodium pyruvate at 33°C in the presence of 5% CO_2_. For confocal microscopy, cells were plated (2,000/well) in 97 glass bottom plates and on the next day transfected with eGFP-tagged constructs utilizing Lipofectamine 2000 Reagent (Invitrogen) following the manufacturer's recommendations for 96 tissue culture plates.

HEK293t cells were cultured in 5% FBS-supplemented DMEM media at 37°C and 5% CO_2_. The overexpression of HTT171 fragments was achieved using Lipofectamine 2000 Reagent. Cells were transfected the day after plating at a seeding density of 6 × 10^4^/ml on 10 cm^2^ dishes. Depending on the expected yield for each vector, three to six tissue dishes were transfected and incubated for 48 h prior to cell harvesting for mitochondrial isolation. Whole cells harvested for SDS-PAGE and immunoblotting were thoroughly washed with PBS before RIPA buffer lysis.

Induced pluripotent stem cells used were control line ND42242 (CAG repeat lengths of 21 and 18) and HD line ND42222 (CAG repeat lengths of 109 and <25) obtained from the NINDS repository. They were propagated as previously described (Singh et al., 2021).

### Animals

C57Bl6 mice were obtained from breeding, with colony founders originally obtained from The Jackson Laboratory. No procedures were performed on live animals in this study. Tissues were obtained from mice of either sex after death for analysis.

### Mitochondrial fractionation

Mitochondria from HEK293t and ST-Hdh-Q7/Q7 cell lines were isolated using Mitochondria Isolation MACS Kit (Miltenyi Biotec) which enables us to get extra pure organelles based on pulling down mitochondria from suspension by anti-TOM22 antibody conjugated to magnetic nanobeads. Detailed procedure for isolation and validation of the procedure for mitochondrial enrichment is described in Yablonska et al. (2019).

Nonsynaptosomal mitochondria were isolated from forebrains of 3–6-week-old wild-type C57Bl6 mice by differential centrifugation in the Percoll gradient as described in Yano et al. (2014). Freshly purified and functional mitochondria were used in import experiments immediately. Frozen brain tissue of 2.5-month-old BACHD and BACDH-SD mice, generously provided by Dr. Yang, was used for mitochondrial isolation and subcellular fractionation.

### Expansion microscopy

Mitochondria isolated from HEK293t cells 48 h post-transfection were placed on the coverslips and treated following the expansion procedure and imaging as described in Tillberg et al. (2016) and Suofu et al. (2017). The mitochondrial outer membrane was detected with anti-TOM20 antibody and the inner membrane with anti-ATP5A antibody. The localization of HTT 171-aa-long was detected with anti-FLAG antibody and 17 aa fragment with anti-eGFP antibody.

### Confocal microscopy and imaging

The fluorescence imaging of cells was performed using an IX-81-DSU Olympus confocal microscope equipped with CCD camera Orca-R2 (Hamamatsu Photonics), Lumen 200 fluorescence illumination system, and motorized XY-precision position flat top stage H117 (Prior Scientific). To provide an optimal environment for cells during long-term live imaging, the microscope was equipped with a WSKM incubation system that controls temperature and gas composition.

The microscope settings for multiwavelength fluorescent imaging were the following: excitation (emission) wavelength, 488 (520) nm for eGFP; 560 (590) nm for TMRM; 644 (665) nm for MitoTracker Deep Red; and 350 (461) nm for Hoechst 33342 nuclear staining dye 350 (461). MitoTracker Deep Red (Invitrogen) and Hoechst 33342 staining dye (Invitrogen) were added to the cell culture 1 h prior to imaging and in concentration range following the manufacturer’s recommendations.

Super-resolution live imaging was performed using laser scanning confocal microscope Olympus FV3000RS equipped with FV-OSR (Olympus Super Resolution) software module allowing to improve resolution to 120–180 nm (XY plane).

### Trypsin treatment

Twenty micrograms of mitochondria isolated from HEK293t cells in 20 µl reaction mixture volume were subjected to trypsin (25 µg/ml) or trypsin and digitonin (0.25%) treatment for 15 and 30 min. The digestion reaction was stopped by adding protease inhibitors cocktail; mitochondrial samples were lysed with RIPA buffer and subjected to immunoblotting for HTT171 constructs and proteins of different mitochondrial compartments [outer mitochondrial membrane (OMM), MIM, and matrix].

### Coimmunoprecipitation

Human iPS cells were lysed in ice-cold TNE immunoprecipitation buffer (10 mM Tris, 150 mM NaCl, 1 mM EDTA, 1% NP40, pH 8) supplemented with a protease inhibitor cocktail to generate 600 μg of protein lysate. In order to lyse cells, a syringe was used to homogenize cells, and three freeze–thaw cycles were performed to permeabilize mitochondrial membranes. Anti-HTT antibody (Abcam, EPR5526, 2.5 μg) was used to immunoprecipitate HTT during overnight incubation at 4°C. Equivalent normal rabbit IgG was used as a control. The Dynabeads Protein G kit (Thermo Fisher Scientific) was used to purify immunoprecipitated components from the cell lysate. The final eluates were separated by SDS-PAGE and immunoblotted for TIM23 and HTT (Yablonska et al., 2019).

### Immunoblotting

A detailed procedure of immunoblotting protocol is described in our publication (Yablonska et al., 2019) in brief, and samples subjected to SDS-PAGE were transferred onto PVDF membranes and immunoblotted with primary antibodies as indicated. The secondary antibody utilized to detect primary antibodies was IRDye infrared fluorescent dye-labeled and detected with Odyssey CLx near-infrared fluorescence imager (LI-COR Biosciences). The intensity of signal was analyzed in Image Studio Version 2.1 software. For presentation purposes, images were converted to monochrome. Fluorescence intensity is represented in graphs as relative to control, with the appropriate control described in each figure legend.

### Surface plasmon resonance Biacore analysis

The experiments to test affinity of protein interaction were performed using a Biacore 1000 instrument (GE HealthCare), and the procedure, reagents, and software used for analysis are described in detail in Yablonska et al. (2019) where binding affinity of HTTexon1-Q23 and HTTexon1-Q97 with TIM23 subunits was investigated and reported. Briefly, anti-GST antibody (30 μg/ml) was coupled to the surface of Series S CM5 sensor chips (GE HealthCare), and unoccupied high-affinity binding sites were blocked with GST proteins. The examined GST-HTT exon1 proteins, each an immobilized ligand, were injected to enable binding with GST antibody. Purified TIM23, TIM50, TIM17A, and TIM17B proteins at different concentrations (0.2, 0.6, 1.8, 5.4, 16.2, and 48.6 nM) were injected into the immobilized ligand surface of the sensor chip to obtain surface plasmon resonance (SPR) sensograms. The baseline-corrected sensograms (with the buffer blank run further subtracted) were globally fitted to a predefined binding model using BIAevaluation software (version 2.0.4).

### Mitochondrial protein import assay

The effect of recombinant mHTT fragment (HTTexon1-Q97 and HTTexon1-Q97-S13ES16E) on mitochondrial protein import was tested using an assay originally outlined in Terada et al. (1997) and described in detail in our previous study (Yablonska et al., 2019). Briefly, pGEM-3Zf(+)-pOTC vector encoding human ornithine transcarbamylase (OTC) precursor (pOTC) was utilized for transcription and translation of [^35^S]-labeled recombinant protein in vitro using TNT T7 Quick Coupled Transcription/Translation System (Promega). The radioactively labeled pOTC and mature cleaved (mOTC) forms were visualized by exposing dried SDS-PAGE gel to phosphor imaging Screen-K (Kodak) and scanned on the Personal Molecular Imager System (Bio-Rad). The intensity of mOTC bands was measured in Image Studio Version 2.1 software, and kinetic curves were plotted based on values quantified from a calibration curve built on mOTC loading.

### LDH assay

The release of LDH was measured from ST-Hdh-Q7/Q7 cells transfected with plasmid vector expressing eGFP-tagged HTT171-Q68 or HTT171-Q68-S13ES16E proteins and sorted with a BD FACS Aria IIu hood to collect eGFP-positive cells 48 h post-transfection. Sorted cells were replated (10,000/well) to 96-well plates in DMEM media supplemented with 5% FBS and streptomycin/penicillin to prevent contamination. The next day, cells were washed with PBS, and media was replaced with serum-deprived DMEM. Twenty-four hours later, cells were subjected to LDH assay according to the manufacturer's instructions of Cytotoxicity Detection Kit^PLUS^ (LDH; Roche).

### Experimental design and statistical analysis

For comparisons between two groups, as in Figures 2, 3*B*, 4*B*, and 6*B*,*E*, two-tailed *t* tests were used to find the statistical significance of differences between samples. For comparisons among multiple groups in Figures 3*E* and 4*E*, we used ANOVA followed by Tukey for pairwise multiple comparisons. For Figure 5*D*, we used a generalized estimating equation model (GEE) for repeated measure analysis. For Figure 2, to exclude potential variability caused by different huntingtin expression, fluorescence intensities of GFP localized to mitochondria (Fig. 2*A*) or nucleus (Fig. 2*B*) were adjusted to the intensity of GFP in cytoplasm for the same cell before statistical analysis. Finally, to accommodate normal biologic variability between sorted cell cultures in Figure 6*D*, a paired *t* test was used, followed by Bonferroni’s correction to control for multiple comparisons. *p* < 0.05 was used as the significance threshold.

## Results

### Phosphomimetic modifications to the N-terminal region of HTT affect its subcellular localization

**Figure 1.**
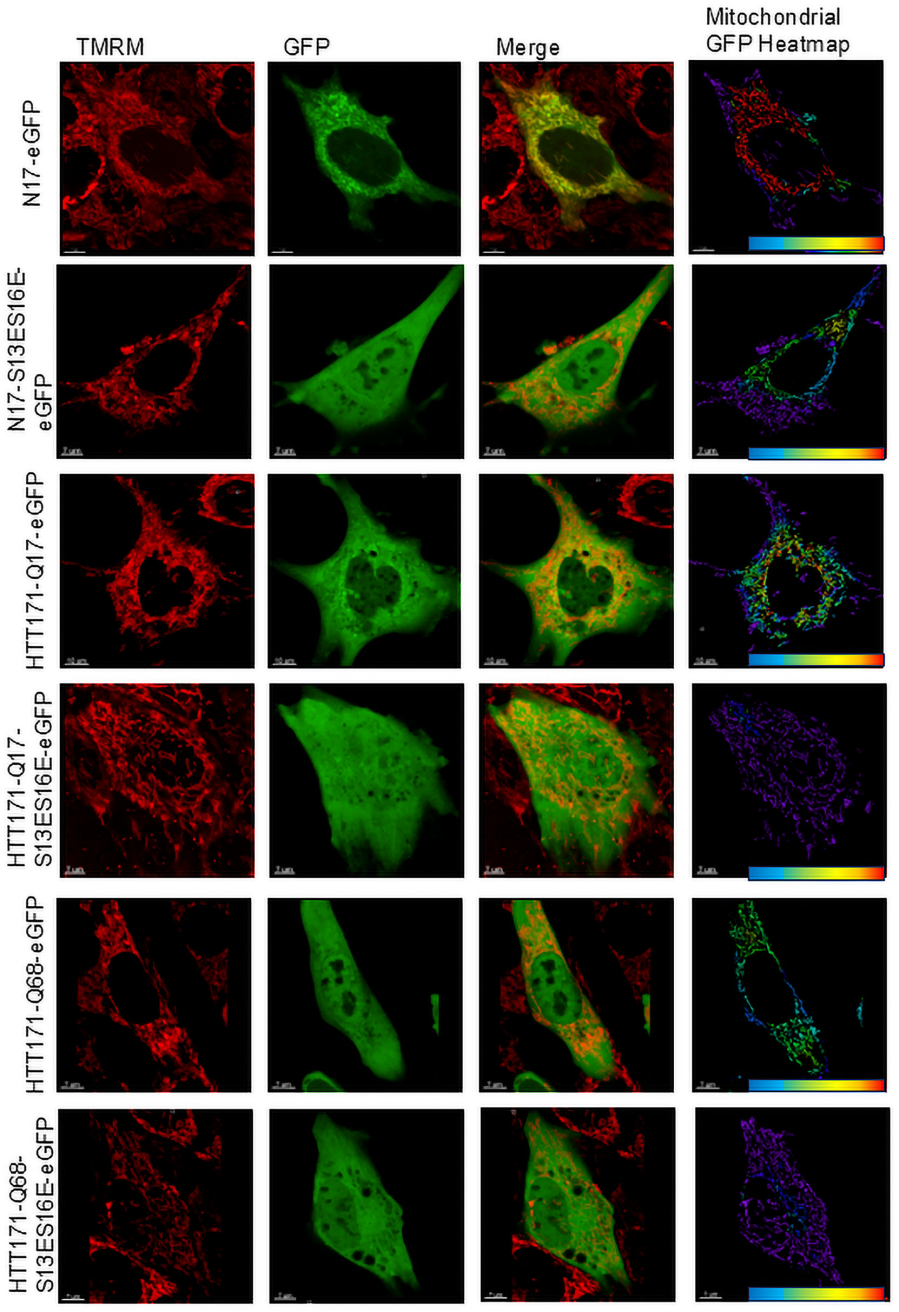
Heatmap of GFP-labeled HTT overlapping with mitochondria. Confocal (Olympus super-resolution mode) live images of cells expressing eGFP-tagged N17 as well as Q17 and Q68-polyQ amino acids long HTT fragments and mitochondria visualized with TMRM. Overlap of eGFP and TMRM signal in each mitochondrial volume was quantitated as the mean intensity of the eGFP signal localized to TMRM only; results were presented as a mitochondrial HTT heatmap. Then, each eGFP over TMRM signal intensity was normalized by eGFP signal found outside of mitochondrial volumes. Thus, the normalization accounts for a difference in eGFP-tagged HTT fragment expression levels in different cells (results are presented in Fig. 2). In the heatmap, red represents a strong overlap of GFP and TMRM and indicates close colocalization of these two signals; color transformation to yellow and green is due to lesser colocalization of GFP and TMRM, and purple indicates that the signals do not overlap. Representative images of the experiment, performed in triplicate, are shown in the figure. ST-Hdh-Q7/Q7 controls are presented in Extended Data Figure 1-1.

**Figure 2.**
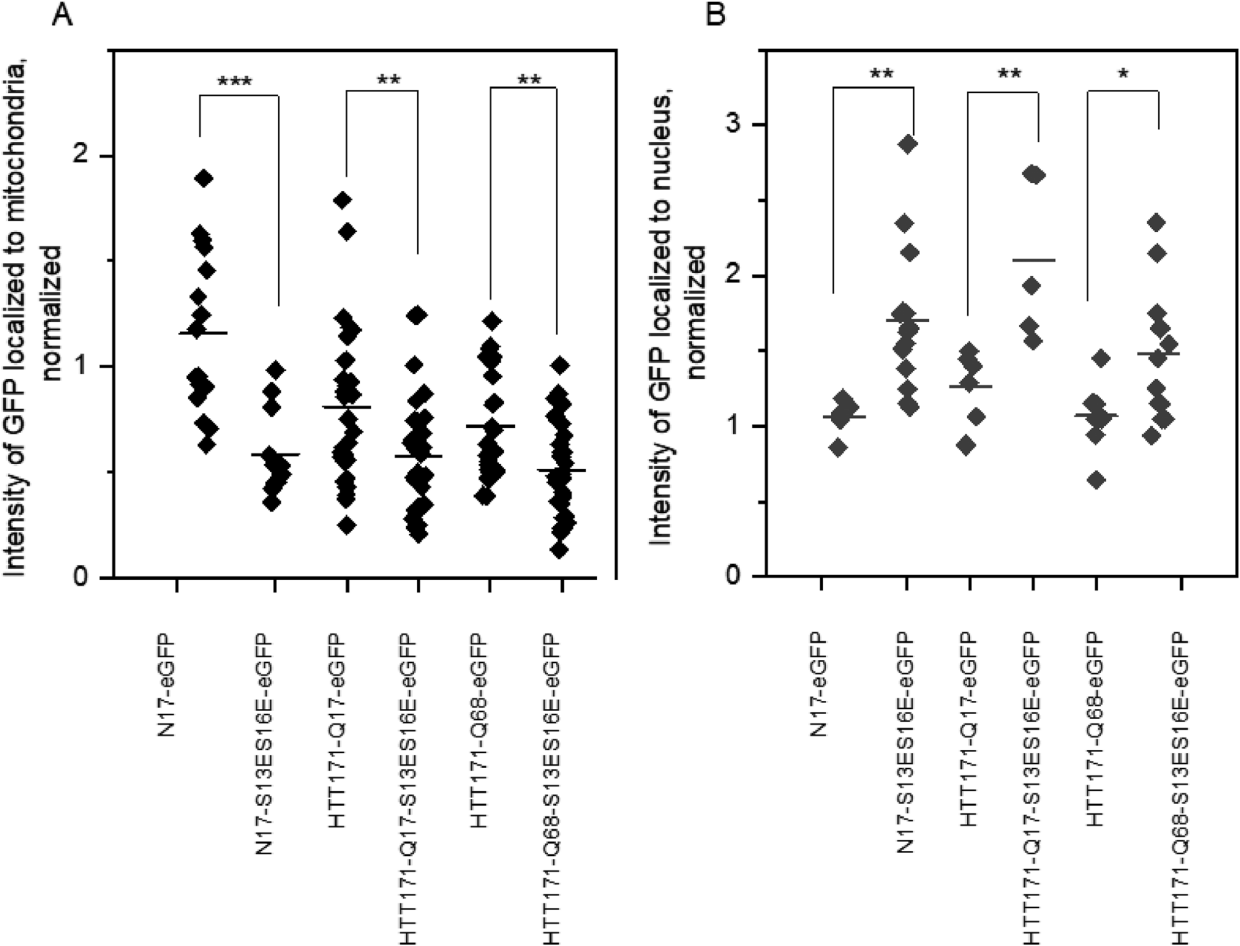
Mitochondrial (***A***) and nuclear (***B***) analysis of eGFP-tagged HTT. Each dot on the graph represents the averaged eGFP signal of N17 and N17-S13ES16E, HTT171-Q17 and HTT171-Q17-S13ES16E, and HTT171-Q68 and HTT171-Q68-S13ES16E in the mitochondria (***A***) and the nucleus (***B***). To account for the difference in the expression level of eGFP-tagged HTT fragments, the mitochondrial and nucleus-associated eGFP signals were normalized to cytoplasmic eGFP signal intensity. Imaging was done on ST-Hdh-Q7/Q7 cells. Results of a one-way ANOVA followed by a posttest for at least three independent sets of collected data are shown on the graphs (*p < 0.05, **p < 0.01, ****p < 0.0001).

The 17 aa HTT N-terminal sequence (N17) forms an amphipathic α-helix that enables HTT to associate with membranes and is known to affect subcellular localization and degradation of HTT (Atwal et al., 2007, 2011; Thompson et al., 2009; Michalek et al., 2013); thus, we started by visualizing the intracellular distribution of HTT. We used eGFP-tagged constructs transfected into mouse striatal cells expressing wild-type *Htt* with seven CAG repeats (ST-Hdh-Q7/Q7) for super-resolution live microscopy. We fused eGFP with the first 171 (HTT171) amino acids of HTT and with N17 itself. To visualize and quantify the mitochondrial and nuclear distribution of HTT171 and N17, we used TMRM (Fig. 1) and DAPI (Extended Data Fig. 1-1), respectively. We did not observe any visible alterations in the mitochondrial network as a result of transfection; nontransfected mitochondrial morphology was not different from transfected mitochondrial morphology. The amount of colocalized eGFP-fused HTT with mitochondria is shown as an eGFP signal intensity heatmap colocalized with mitochondria (Fig. 1, fourth column). N17-eGFP and both wild-type (Q17) and mutant (Q68) HTT171 colocalize with mitochondria. Interestingly, N17-eGFP alone had shown the strongest mitochondrial targeting, suggesting the additional 154 HTT amino acid residues alter the subcellular targeting. Phosphomimetic mutations in N17 disrupt its helical conformation (DeGuire et al., 2018) resulting in nuclear translocation of HTT fragments (Atwal et al., 2011). When we replaced the 13th and 16th serine of HTT with the phosphomimetic amino acid residue (glutamic acid; S13ES16E), we observed a reduction of S13ES16E mutants’ accumulation in mitochondria (Figs. 1, 2*A*) and reciprocal increase of nuclear colocalization (Fig. 1, 2*B*; Extended Data Fig. 1-1). Therefore, the N17 region of HTT plays a key role in determining subcellular localization, and phosphomimetic changes reduce mitochondrial and increase nuclear localization.

10.1523/JNEUROSCI.1254-24.2024.f1-1 Figure 1-1 Nuclear staining (DAPI-blue) of ST-Hdh-Q7/Q7 cells expressing phosphomimetic (S13ES16E) and non-mutated counterparts of N17-eGFP, HTT171-Q17-eGFP and -Q68-eGFP constructs. Representative images of super resolution microscopy showing distribution of eGFP signal. Download Figure 1-1, TIF file.

### N17 phosphomimetic modulates HTT entry into the mitochondria

**Figure 3.**
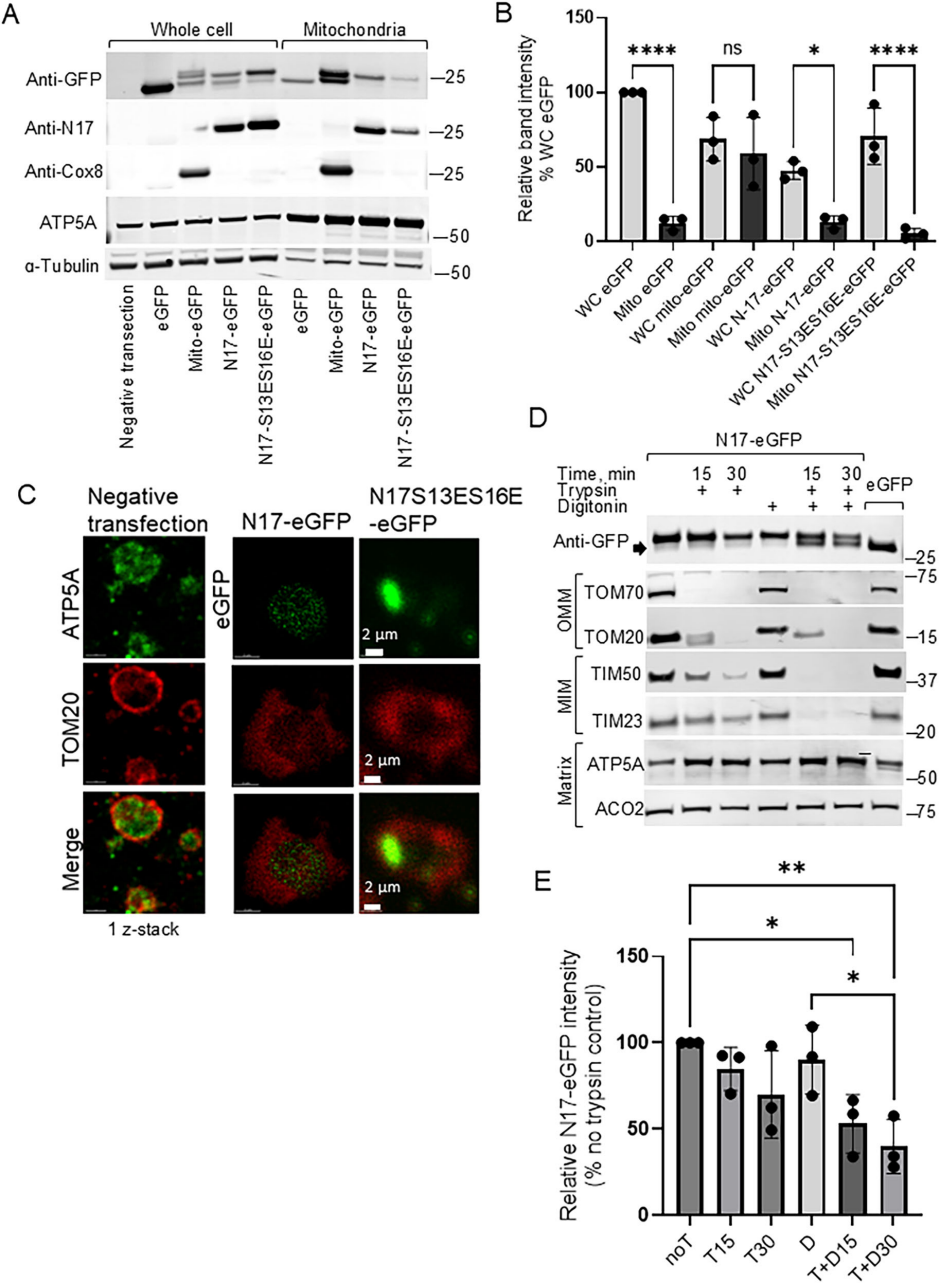
N-terminal amino acids of HTT function as mitochondrial targeting signal. Representative immunoblot (***A***, uncropped immunoblots; Extended Data Fig. 3-1) and immunoblot quantification (***B***) demonstrate levels of N17-eGFP, N17-S13ES16E-eGFP, mito-eGFP, and eGFP proteins in mitochondrial fractions and whole cell lysate of HEK293t cells. Cells were harvested for mitochondrial isolation 48 h post-transfection. N.t., negative transfection sample; eGFP, plain fluorescent protein, serves as a nontagged control. Mito-eGFP serves as control for mitochondrial colocalization due to COX8A mitochondrial targeting signal. The expression of all proteins was detected with anti-GFP antibody, anti-HTT antibody detects only the N-terminal HTT sequence, and the anti-COX8A antibody detects only the mitochondrial targeting sequence of COX8A. ATP5A and α-tubulin serve as loading control. For quantification (***B***), the intensity of each anti-eGFP detected protein band was normalized to ATP5A level in the same sample. For relative protein quantification, protein quantification is expressed relative to whole cells (WC) eGFP. Analysis of the mitochondrial (mito) eGFP was compared with WC for each sample type using one-way ANOVA followed by Sidak's test for multiple comparisons (black bars on a graph; *n* = 3, data shown as mean ± SEM, ***p < 0.001, ****p < 0.0001, ANOVA plus Sidak's test). ***C***, Expansion microscopy images show localization of the 17 aa N-terminal HTT sequence within mitochondria detected by anti-eGFP antibody. TOM20 labels the outer mitochondrial membrane, and ATP5A is a control for an endogenous matrix protein in a nontransfected cell. N17-S13ES16E localization is different than N17. Representative immunoblot (***D***, uncropped immunoblot in Extended Data Fig. 3-2) and quantification of the upper band (i.e., uncleaved N17-eGFP; ***E***) demonstrating mitochondrial localization of N17-eGFP, probed with an anti-GFP antibody, in HEK293t mitochondria. The black arrow on the anti-GFP panel indicates the molecular weight shift of the N17-eGFP construct after trypsin digestion which only removes the N17 sequence, since eGFP protein is not digestible at these conditions. T stands for trypsin and D for digitonin (***E***). After blots were stripped, they were probed for mitochondrial subcompartment markers: TOM70 and TOM20 for outer mitochondrial membrane (OMM), TIM50 and TIM23 for mitochondrial inner membrane (MIM), and ATP5A and ACO2 for matrix. Immunoblot bands were normalized to the untreated sample (noT), which was taken as 100% (*n* = 3, data shown as mean ± SEM, *p < 0.05, **p < 0.01, ANOVA plus Tukey). N17-eGFP and mito-eGFP controls are shown in Extended Data Figure 3-3.

To confirm intramitochondrial localization of N17 constructs by immunoblotting, we utilized two techniques, i.e., selective digestion and expansion microscopy, that demonstrate HTT localization in a complementary manner. We transfected HEK293t cells with plasmid vectors and after 2 d isolated mitochondria using magnetic nanobeads. First, we compared the content of N17-eGFP and N17-S13ES16E-eGFP proteins to mito-eGFP, a construct of eGFP fused with the mitochondrial targeting sequence of COX8A, an endogenous mitochondrial protein. Double bands observed for all fused constructs in whole cell lysate (Fig. 3*A*, top panel; Extended Data Fig. 3-1) are the original form of the protein and a cleavage product as detected with an anti-GFP antibody. Anti-HTT, which binds an epitope within N17, and anti-COX8 antibodies detect only one higher size band of the protein due to the presence of a targeting sequence recognized by the antibody (Fig. 3*A*, second and third panels; Extended Data Fig. 3-1). Utilizing these antibodies, we detected N17-eGFP as well as N17-S13ES16E-eGFP in the mitochondrial enriched fraction (Fig. 3*A*, Extended Data Fig. 3-1). Although phosphomimetic N17 had the highest expression compared with the two other fused constructs (mito-eGFP and N17-eGFP) in the whole cell lysate, the amount of N17-S13ES16E in the mitochondria was the lowest and similar to eGFP, suggesting that it might be isolated as a contaminant (Fig. 3*B*). Notably, mito-eGFP had the highest mitochondrial levels among all fused constructs even though total expression level in whole cell lysate was similar to N17-eGFP, indicating high relative mitochondrial targeting of the COX8A sequence as compared with N17. These data show that N17 targets HTT to the mitochondria; however, N17 is a less potent mitochondrial targeting sequence as compared with a classic mitochondrial protein COX8. It is notable that mito-eGFP in the mitochondrial enriched fraction has a high intensity lower molecular weight band, whereas the intensity of the lower band is lower in the N17 constructs. This suggests that as expected the COX8A-linked eGFP protein is transported to the mitochondrial matrix where the mito-targeting signal is cleaved. In contrast, the N17-linked eGFP either does not enter the matrix or is not cleaved.

10.1523/JNEUROSCI.1254-24.2024.f3-1 Figure 3-1 Uncropped immunoblot images for Figure 3a. Download Figure 3-1, TIF file.

10.1523/JNEUROSCI.1254-24.2024.f3-2 Figure 3-2 Uncropped immunoblot images for Figure 3d. Download Figure 3-2, TIF file.

10.1523/JNEUROSCI.1254-24.2024.f3-3 Figure 3-3 N17-terminal amino acid sequence of HTT and mitochondrial targeting sequence of COX8A induce eGFP mitochondrial translocation. (a) Representative immunoblot showing digestion pattern of N17-eGFP (a) and mito-eGFP (b) in mitochondria of HEK293t cells. Mito-eGFP serves as control for intra-mitochondrial/matrix localization due to the canonical COX8A mitochondrial targeting signal. The expression of proteins was detected with anti-HTT antibody that recognizes N-terminal sequence of HTT (a), and anti-COX8A antibody (b). After stripping blots were probed for markers for mitochondrial sub-compartment comparisons: TOM70 for outer mitochondrial membrane (OMM), TIM23 for mitochondrial inner membrane (MIM), and ACO2 for matrix. Cells were harvested for mitochondria isolation 48 hours post-transfection, N.t. – negative transfection sample. Download Figure 3-3, TIF file.

To determine the submitochondrial localization of the N17-eGFP construct, we isolated mitochondria and subjected them to expansion microscopy (Fig. 3*C*) and digestion with trypsin and trypsin in combination with digitonin, which permeabilizes membranes (Fig. 3*D,E*) as described in Yablonska et al. (2019). Mitochondrial expansion microscopy enabled us to visualize submitochondrial compartments in isotropic proportions to localize eGFP-fused constructs. To visualize mitochondria, we labeled their outer and inner membranes by immunostaining TOM20 (Fig. 3*C*, red staining) and ATP5A, respectively (Fig. 3*C*, green staining in the negative transfection sample). The negative transfection sample shows boundaries of the outer membrane and packaging of the inner membrane inside mitochondria. Mitochondria isolated from cells expressing N17-eGFP demonstrate localization of eGFP within boundaries of the mitochondrial outer membrane, whereas N17-S13ES16E-eGFP was seen mostly overlapping the outer membrane (Fig. 3*C*). Therefore, N17 is a “bona fide” mitochondrial targeting sequence and phosphomimetic modifications impair mitochondrial penetration.

The localization of N17-eGFP was determined by comparing its digestion pattern with mitochondrial proteins of known location such as outer membrane TOM70, TOM20, inner membrane TIM50, TIM23, and matrix ATP5A and ACO2 proteins. When solely adding trypsin, proteins on the outer membrane of the mitochondria or external contaminants are digested (TOM70, TOM20), but proteins within the outer membrane cannot be reached. When trypsin and digitonin are added, proteins on the outer membrane, in the intermembrane space, and in the inner membrane (TIM50, TIM23) are digested. eGFP protein does not have trypsin-sensitive digestion sites, so trypsin digestion occurs only in the HTT amino acid sequence. Thus, trypsin digestion of this protein is indicated by the molecular weight shift (Fig. 3*D*, black arrow, probed with an anti-GFP antibody; Extended Data Fig. 3-2). Mitochondrial N17-eGFP is partially digested by trypsin treatment, suggesting that some of the protein is associated with the outer mitochondrial membrane (Fig. 3*D*,*E*; Extended Data Figs. 3-2, 3-3*A*). The remaining N17-eGFP resisted trypsin digestion even in combination with digitonin, which permeabilizes the outer mitochondrial membrane, suggesting matrix or inaccessible inner membrane N17-eGFP localization. The digestion pattern of N17-eGFP (Extended Data Fig. 3-3*A*, probed with anti-HTT antibody that detects only HTT N17 epitope) matched the digestion pattern of mito-eGFP, a control construct that contains the 25-aa-long mitochondrial targeting sequence of COX8, which is a control for a matrix protein (Extended Data Fig. 3-3*B*). Expansion microscopy and immunoblotting data taken together demonstrate that the first 17 aa of HTT contain targeting information for import into mitochondria and make HTT protein a “bona fide” resident of the mitochondria. Furthermore, phosphomimetic mutations reduce the amount of N17 transport into the mitochondria.

### Phosphomimetic mutations of serines 13 and 16 reduce the amount of HTT in the mitochondria

**Figure 4.**
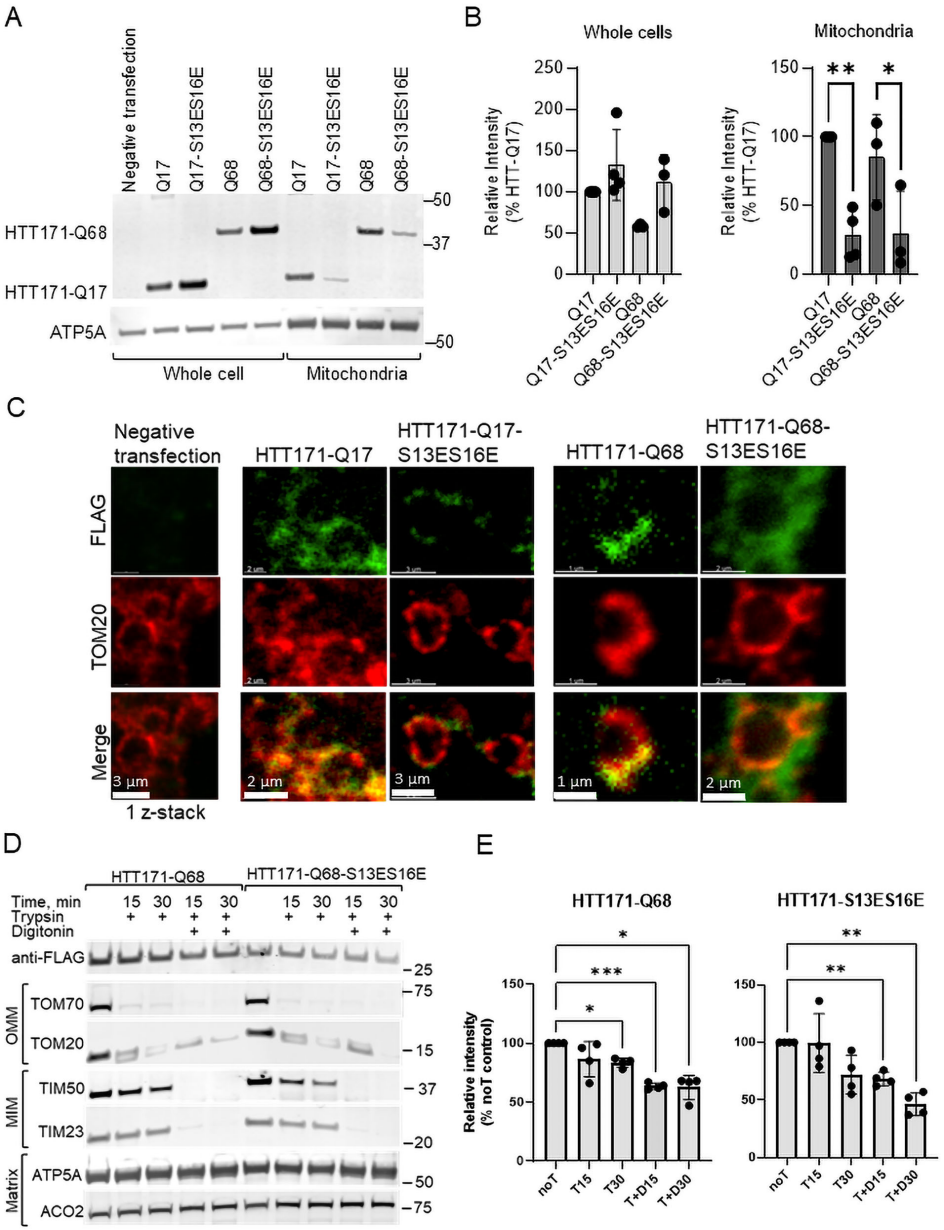
The phosphomimetic mutations reduce the amount of HTT fragment in mitochondria. ***A***, Representative immunoblot of whole cell and mitochondrial lysates probed with anti-FLAG antibody for wtHTT and mHTT (HTT171-Q17 and HTT171-Q68) and HTT phosphomimetics (HTT171-Q17-S13ES16E and HTT171-Q68-S13ES16E) levels. ATP5A acts as a loading control for all samples. Uncropped immunoblots are shown in Extended Data Figure 4-1. Q17 and Q17-S13ES16E data are shown in Extended Data Figure 4-2. ***B***, Quantification of HTT171 fragments in whole cell and mitochondrial lysates, the intensity of each band was normalized to ATP5A level in the same sample and normalized to the control sample band intensity (HTT171-Q17) which was taken as 100% and used to recalculate values for the rest of the samples. N.t., negative transfection sample (*n* = 3–4, data shown as mean ± SEM, *p < 0.05, ***p < 0.001, two-way ANOVA plus Tukey). ***C***, Imaging of expanded mitochondria isolated from HEK293t cells transfected with HTT171 fragments. Staining for TOM20 shows the outer mitochondrial membrane (red), and green staining represents the detection of FLAG-tag of HTT171-Q17 and -Q68 fragments and phosphomimetic versions. Representative immunoblot (***D***, uncropped immunoblots in Extended Data Fig. 4-3) and quantification (***E***) demonstrating localization in mitochondria of mHTT phosphomimetic (HTT171-Q68-S13ES16E) comparing to nonmutated counterpart (HTT171-Q68). Expression of both proteins in HEK293t mitochondria was detected with an anti-FLAG antibody. T stands for trypsin and D for digitonin (***E***). After stripping blots were probed for markers for mitochondrial subcompartment comparisons: TOM70 and TOM20 for OMM, TIM50 and TIM23 for MIM, and ATP5A and ACO2 for matrix. Immunoblot bands were normalized to the untreated sample (noT), which was taken as 100% (*n* = 4, data shown as mean ± SEM, *p < 0.05, ***p < 0.001, ****p < 0.0001, ANOVA plus Sidak).

To test whether the phosphomimetic substitutions of amino acids in the N17 sequence are important for mitochondrial targeting of HTT, we utilized the 171-aa-long fragment of HTT with two phosphomimetic mutations, serines 13 and 16 (HTT171-Q17-S13ES16E and HTT171-Q68-S13ES16E). The Q17 and Q68 constructs are HTT fragments with 17 and 68 CAG repeats (encoding for glutamine) representing wild type and mHTT, respectively. HTT fragments were overexpressed in HEK293t cells and visualized by immunoblotting. The protein expression level of phosphomimetic versions of both wtHTT and mHTT proteins (HTT171-Q17-S13ES16E and HTT171-Q68-S13ES16E on Fig. 4*A*, Extended Data Fig. 4-1) was higher than nonmutated counterparts (Fig. 4*A*,*B*; Extended Data Fig. 4-1), similar to N17-S13ES16E-eGFP. Despite the high expression, the level of phosphomimetic wtHTT fragment in the mitochondria was less than nongenetically altered wtHTT, suggesting that phosphomimetics wtHTT are less efficiently imported into mitochondria despite higher cellular concentrations. Even though the amount of mHTT phosphomimetic in the mitochondria was not statistically lower than the unmodified mHTT fragment, there is a visual difference between them on the representative immunoblot which was highly reproducible.

10.1523/JNEUROSCI.1254-24.2024.f4-1 Figure 4-1 Uncropped immunoblot images for Figure 4a. Download Figure 4-1, TIF file.

10.1523/JNEUROSCI.1254-24.2024.f4-2 Figure 4-2 The phosphomimetic mutations reduce the amount of wtHTT fragment in mitochondria. Representative immunoblot (a) and quantification (b) demonstrating localization in mitochondria of wtHTT phosphomimetic (HTT171-Q17-S13ES16E) comparing to non-mutated counterpart (HTT171-Q17). Expression of both proteins in HEK293t mitochondria was detected with anti-flag antibody. After stripping blots were probed for markers for mitochondrial sub-compartment comparisons: TOM20 for OMM, TIM23 for MIM, and ATP5A, ACO2 for matrix. Immunoblot bands were normalized to the untreated sample (noT stands for no trypsin), which was taken as 100%. Download Figure 4-2, TIF file.

10.1523/JNEUROSCI.1254-24.2024.f4-3 Figure 4-3 Uncropped immunoblot images for Figure 4d. Download Figure 4-3, TIF file.

To determine submitochondrial HTT localization, we performed expansion microscopy, which uses a hydrogel to expand a sample during processing, followed by super-resolution microscopy. Mitochondria isolated from cells expressing mHTT and wtHTT proteins were first stained for TOM20, the outer mitochondrial membrane protein, and then subjected to expansion to enlarge submitochondrial compartments. Both wtHTT and mHTT fragments (green staining for C-terminal FLAG-tag on all four constructs) colocalized with outer the mitochondrial membrane overlapping with TOM20 staining (yellow; Fig. 4*C*), whereas phosphomimetic mutants (S13ES16E) of both wtHTT and mHTT fragments were located on the outer side of membrane not producing yellow overlap signal. We found the incidence of colocalized TOM20 with phosphomimetic mutants to be rare. We explain this as the weak binding of the mutant to the mitochondrial outer membrane, which is easily damaged during mitochondrial isolation.

By comparing the digestion pattern of HTT to mitochondrial proteins with known mitochondrial location, we determined that both mHTT and wtHTT phosphomimetic proteins localize to the same subcompartment, intermembrane space of the mitochondria and matrix; however, the phosphomimetic does so less efficiently (Fig. 4*D*,*E*; Extended Data Fig. 4-1). The analysis of the digested bands of HT171-Q17 and HTT171-Q68 and both phosphomimetic mutants compared with outer membrane proteins (TOM70, TOM20) shows that, on average, 15% of HTT is located on the outer side of the mitochondrial membrane (Fig. 4*E*, Extended Data Fig. 4-2). Another 20% of HTT is located in the mitochondrial intermembrane space since it is getting cleaved in samples treated by a combination of trypsin and digitonin (Fig. 4*D*,*E*; Extended Data Fig. 4-3), similar to TIM23 and TIM50. However, a sizable portion of HTT171 (62% of mHTT in the mitochondria after trypsin and digitonin treatment compared with 46% of phosphomimetic mHTT) remains nondigested, suggesting an inaccessible inner mitochondrial membrane or matrix localization of HTT which is not observed on expansion samples. This might be due to the inaccessibility of FLAG epitope on expanded mitochondria or wash out of these proteins during the expansion process. Together our data demonstrate that both wtHTT and mHTT are intramitochondrial proteins and phosphomimetic modifications to N17 reduce HTT mitochondrial targeting.

### Phosphomimetic mutations ameliorate mHTT's effect on mitochondrial function

**Figure 5.**
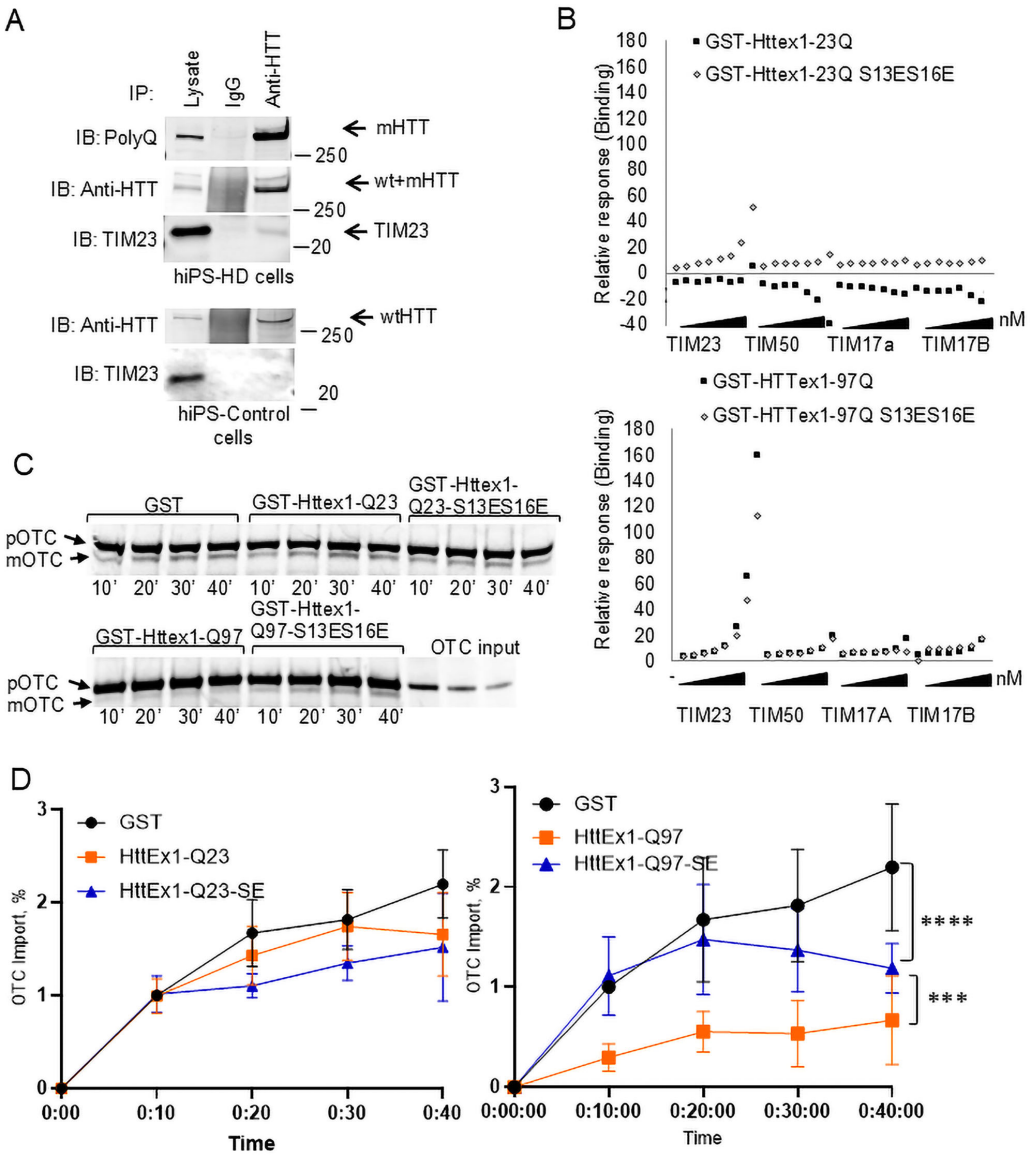
mHTT phosphomimetic shows lower binding affinity with TIM23 and causes lesser inhibition of mitochondrial protein import function. ***A***, Full-length endogenous mHTT directly interacts with the TIM23 complex. Immunoblot image of full-length HTT coimmunoprecipitation with an anti-HTT antibody EPR5526 in human iPS cells [the top panel shows HD iPS cells (109 CAG), and the bottom panel shows non-HD control iPS cells]. Full-length mHTT was immunoprobed with an anti-polyQ antibody; both wtHTT and mHTT were immunoprobed with an anti-HTT antibody EPR5526. TIM23 was detected with an anti-TIM23 antibody. Normal rabbit IgG was used as a negative control. Uncropped immunoblots in Extended Data Figure 5-1. ***B***, Previously published representative sensogram showing binding affinity of TIM23 complex subunits to HTTexon1-Q23 and HTTexon1-Q97 (Yablonska et al., 2019) compared with previously unpublished -Q23-S13ES16E and -Q97-S13ES16E. The *x*-axis represents the concentration range each subunit was tested (from 0.2 to 48.6 nM) depicted by a triangle for each subunit. Raw sensograms (Extended Data Fig. 5-2*A–D*) were analyzed to extract relative response values for each subunit (TIM23, TIM50, TIM17A, TIM17B), which were separately tested at the concentration range of 0.2–48.6 nM (Extended Data Table 5-1). Representative SDS-PAGE (***C***, uncropped immunoblots in Extended Data Fig. 5-3) and kinetic analysis (***D***) show lesser inhibition of mitochondrial [^35^S]OTC import, as measured as accumulation of the lower mOTC band. Forebrain mitochondria prepared from adult wild-type mice were preincubated with recombinant GST-HTTex1 and GST or proteins (150 nM) on ice for 1 h and then subjected to import assays for 10, 20, 30, and 40 min. pOTC, premature form of the protein; mOTC, mature cleaved form (*n* = 3, data shown as mean ± SD. Asterisks indicate a difference in total protein accumulation. *p < 0.05, ***p < 0.001, ****p < 0.0001, GEE for repeated measures).

mHTT N17 is not only a key to its subcellular localization (Rockabrand et al., 2007). We found that these amino acids are also essential for mHTT translocation and binding to the TIM23 subunit of the TIM23 mitochondrial protein import complex. We previously demonstrated that the N-terminal mHTT fragment binds to TIM23 with high affinity [equilibrium dissociation constant (KD) = 5.05 × 10^−13^], while wtHTT did not bind to TIM23 (Yano et al., 2014; Yablonska et al., 2019). Given that we previously demonstrated mHTT–TIM23 binding using mouse mitochondria and HTT fragments, we sought to determine whether this interaction also occurs in human cells expressing endogenous full-length HTT. We confirm that endogenous full-length mHTT and TIM23 bind in HD patient induced pluripotent stem (hiPS-HD) cells (NINDS repository line NS000143). We utilized an anti-HTT antibody to immunoprecipitate full-length HTT in hiPS-HD and control human induced pluripotent stem (hiPS-control) cells. mHTT coimmunoprecipitates with TIM23 (Fig. 5*A*, Extended Data Fig. 5-1), whereas no detectable TIM23 interaction is detected with wtHTT in hiPS-control cells.

10.1523/JNEUROSCI.1254-24.2024.t5-1 Table 5-1 Binding affinity constants of TIM23 complex subunits with mHTT exon1. Download Table 5-1, DOCX file.

10.1523/JNEUROSCI.1254-24.2024.f5-1 Figure 5-1 Uncropped immunoblot images for Figure 5a. Download Figure 5-1, TIF file.

10.1523/JNEUROSCI.1254-24.2024.f5-2 Figure 5-2 Representative SPR sensorgrams displaying binding of TIM23, TIM50, TIM17A and TIM17B proteins (sample) to immobilized ligand wild type HTT (HTTex1-23Q) (a), wild type HTT phosphomimetic (GST-HTTex1-23Q-S13ES16E) (b), mutant HTT (GST-HTT-97Q) (c) and mutant HTT phosphomimetic (GST-HTT-97Q-S13ES16E) (d). Download Figure 5-2, TIF file.

10.1523/JNEUROSCI.1254-24.2024.f5-3 Figure 5-3 Uncropped immunoblot images for Figure 5c. Download Figure 5-3, TIF file.

It is of interest that phosphomimetic substitutions of serine residues at HTT positions 13 and 16 with glutamic (E) or aspartic acid (D) completely abolish mHTT-mediated toxicity in vitro (Atwal et al., 2011) and in vivo (Gu et al., 2009). Therefore, we evaluated whether S13ES16E substitutions in mHTT and wtHTT altered the binding affinity to the TIM23 complex. We previously performed and published affinity binding assays with purified recombinant wtHTT (Httex1-23Q) and mHTT (Httex1-Q97) with TIM23 complex subunits and found that mHTT binds TIM23 subunit with higher affinity than wtHTT (Yablonska et al., 2019). In this study, we compare these previously published data with phosphomimetic counterparts (Httexon1-23Q-S13ES16E, Httexon1-97Q-S13ES16E) and individual TIM23 complex subunits using a surface plasmon resonance (SPR) Biacore analysis platform (Extended Data Fig. 5-2, Extended Data Table 5-1). We note that although the Httex1Q23 and HttQ97 data were previously published, the Httexon1-23Q-S13ES16E and Httexon1-97Q-S13ES16E samples were tested at the same time but not reported previously (Yablonska et al., 2019). The HTT exon1-97Q-S13ES16E protein showed a 30% reduced binding affinity to TIM23 subunit compared with its HTTexon1-97Q counterpart protein (Fig. 5*B*). Other tested subunits, TIM50, TIM17A, and TIM17B, did not show significant association with either HTTexon1-97Q or -97Q-S13ES16E. Interestingly, wtHTT phosphomimetic showed binding to TIM23, but the biological relevance (if any) is unclear.

Binding and inhibition of mitochondrial protein import is an early event in HD pathogenesis (Yano et al., 2014). We previously demonstrated mHTT-mediated inhibition of mitochondrial protein import (Yano et al., 2014). Given the reduced binding affinity and reduced mitochondrial penetration of phosphomimetic mHTT, we evaluated whether this modification alters the ability of mHTT to inhibit mitochondrial protein import (Yano et al., 2014). We show that purified mHTT fragments inhibit protein import in isolated mitochondria (Yano et al., 2014) utilizing an established assay with [^35^S]-labeled mitochondrial matrix enzyme pre-ornithine transcarbamylase (pOTC; Terada et al., 1997). Upon incubation with mitochondria, pOTC is imported into the matrix where the cleavage of the N-terminal targeting sequence results in generation of shorter mature OTC (mOTC). Recombinant HTT proteins were incubated with fresh nonsynaptosomal mitochondria isolated from wild-type mice brains for 1 h to enable protein interaction with importing complexes. After the pOTC import reaction was stopped, we separated the reaction mixture on SDS-PAGE, and in addition to the expected premature OTC (pOTC) band, we detected a lower mOTC band which indicates a cleavage of imported labeled recombinant protein in mitochondria. Both mHTT and S13ES16E mHTT decrease the total amount of OTC imported by mice forebrain mitochondria (Fig. 5*C*,*D*; Extended Data Fig. 5-3). In keeping with the hypothesis, however, the total amount of imported OTC is higher in the presence of S13ES16E mHTT than in mHTT without the phosphomimetic mutation. Therefore, improved mitochondrial protein import is concordant with the reduced affinity of S13ES16E mHTT for TIM23 and reduced mitochondrial targeting, suggesting a crucial role of the N17 domain in mHTT-mediated alteration of the mitochondrial proteome and resultant mitochondrial toxicity (Yano et al., 2014; Yablonska et al., 2019). Therefore, the combination of reduced mitochondrial targeting and reduced binding affinity of N17 phosphomimetic results in a significant reduction of mHTT-mediated mitochondrial and cellular toxicity.

### Phosphomimetic substitutions reduce mHTT-mediated cell death

**Figure 6.**
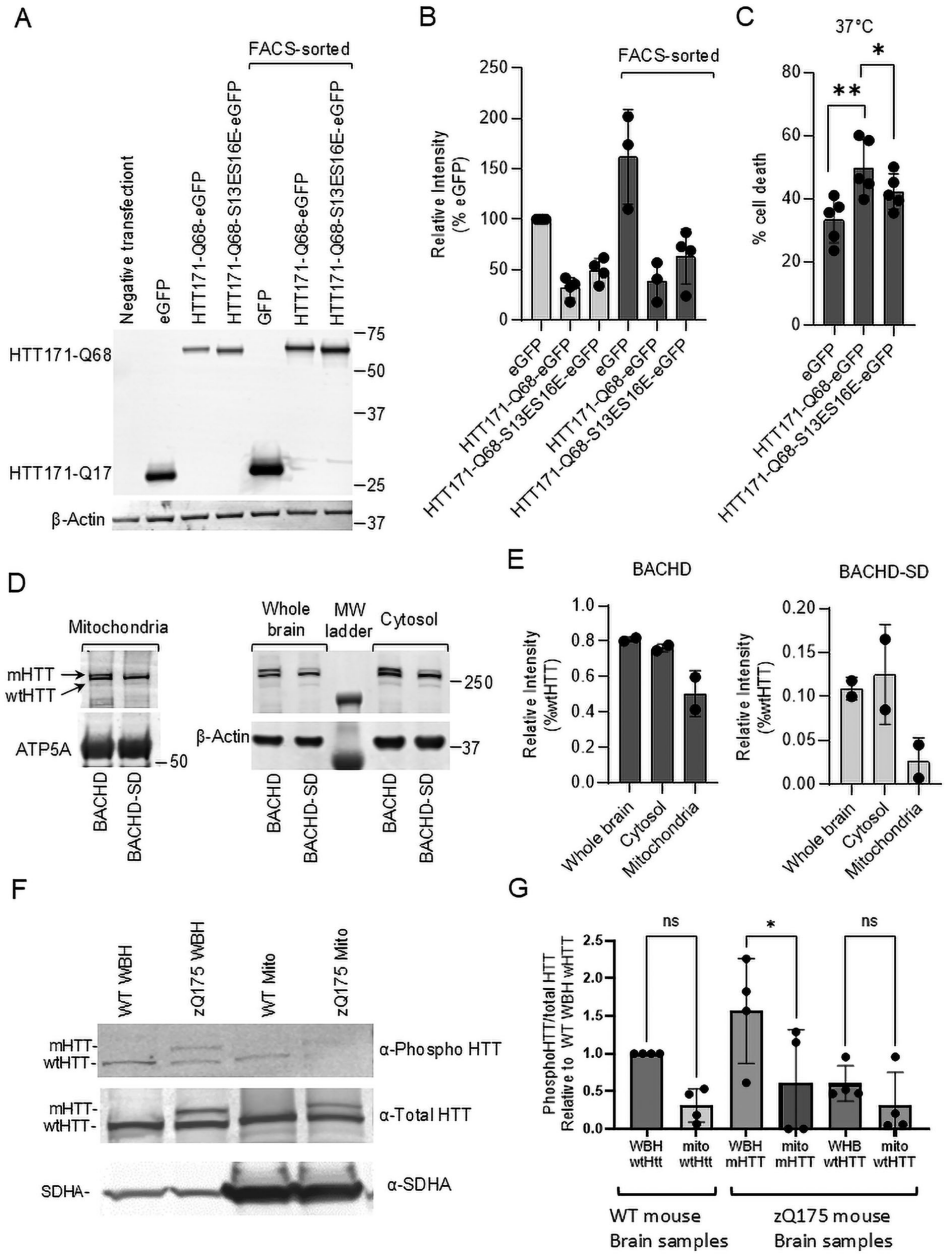
Phosphomimetic mutation reduces mHTT levels in brain mitochondria and modifies mHTT toxicity. ***A***, Expression level of eGFP, HTT171-Q68-eGFP, and -Q68-S13ES16E-eGFP in ST-Hdh-Q7/Q7 striatal cells 48 h post-transfection subjected to FACS sorting of eGFP-positive cells. HTT171 constructs were immunoprobed with anti-eGFP antibody (***A***, uncropped immunoblots in Extended Data Fig. 6-1); loading was controlled by probing for β-actin. The bar graph shows quantified and normalized to β-actin level of HTT171 proteins in cells before and after FACS sorting (***B***). ***C***, Percentage of cell death measured by the release of LDH into the extracellular environment 48 h after eGFP-positive FACS-sorted cells were plated and incubated at 37°C in nonpermissive (serum free) conditions (full FACS data in Extended Data Fig. 6-2). Percentage of cell death in eGFP samples was taken as 100% and used to recalculate values for the rest of the samples (*n* = 5, data shown as mean ± SEM, *p < 0.05, **p < 0.01, paired *t* test plus Bonferonni). Representative immunoblot (***D***, uncropped immunoblots in Extended Data Fig. 6-3) and quantification (***E***) of full-length mHTT and mHTT phosphomimetic in nonsynaptosomal mitochondria, cytosol, and whole brain homogenate of 2.5-month-old transgenic BACHD and BACHD-SD mice. The full-length HTT immunoblotted with anti-HTT antibody MAB2166; mitochondrial ATP5A was probed to ensure equal loading. ***D***, The level of transgenic full-length mHTT harboring two phospho-mutations was normalized to endogenous wild-type HTT in all fractions [two biological repeats each containing five pooled mouse brains (pooling required to be able to isolate sufficient mitochondria); data shown as mean ± SEM]. ***F***, ***G***, Whole brain homogenates (50 μg total protein) and isolated mitochondria (150 μg total protein) were obtained from wild-type (WT) and zQ175KI (zQ) mice and immunoblotted using an S13 phospho-specific HTT antibody, reprobed using a total HTT antibody and then with SDHA antibody for mitochondrial loading. Representative phospho-HTT blot (***F***, uncropped immunoblots in Extended Data Fig. 6-4), and total HTT blot had the fluorescent signals for each band quantified and (***B***) expressed as the ratio of phospho-HTT/total HTT. wtHtt stands for wild-type HTT on the bar graph ***G***. *N* = 4 analyzed by one-way ANOVA plus Sidak's test for multiple comparisons. *p < 0.05.

Next, we evaluated phosphomimetic mHTT construct toxicity as compared with unmodified mHTT. We transfected ST-Hdh-Q7/Q7 striatal cells with HTT171-Q68-eGFP or HTT171-Q68-S13ES13E-eGFP, and eGFP control, and evaluated mHTT-induced cell death by measuring LDH release. We used FACS to obtain a homogenous cell population expressing mHTT or mHTT phosphomimetic and replated them to grow for 48 h in nonpermissive temperature (37°C) and serum-deprived conditions. Released LDH levels were higher in HTT171-Q68-eGFP–expressing cells suggesting more cell death as compared with control eGFP-expressing cells. Although HTT171-Q68-S13ES16E-eGFP protein expression level was at least equal to HTT171-Q68-eGFP (Fig. 6*A*,*B*; Extended Data Fig. 6-1), a lower percentage of cell death was observed in cells expressing phosphomimetic mHTT, 42% in comparison with 50%, a decrease of 16% (Fig. 6*C*). Notably, the percentage of eGFP-positive cells detected by FACS in HTT171-Q68–expressing cells was reproducibly lower compared with population of cells expressing HTT171-Q68-S13ES16E or eGFP [population 4 (P4) value in the table; Extended Data Fig. 6-2]. This suggests higher cell death induced by mHTT expression even prior to FACS. These data provide direct evidence that phosphomimetic mHTT substitutions result in decreased cellular toxicity as compared with mHTT.

10.1523/JNEUROSCI.1254-24.2024.f6-1 Figure 6-1 Uncropped immunoblot images for Figure 6a. Download Figure 6-1, TIF file.

10.1523/JNEUROSCI.1254-24.2024.f6-2 Figure 6-2 Flow cytometry charts of eGFP positive populations of ST-Hdh-Q7/Q7 cells collected 48  h post-transfection expressing eGFP (a), HTT171-Q68 (b) or HTT171-Q68-S13ES16E-eGFP (c). Cells were re-plated immediately into 96-well plates. Download Figure 6-2, TIF file.

10.1523/JNEUROSCI.1254-24.2024.f6-3 Figure 6-3 Uncropped immunoblot images for Figure 6d. Download Figure 6-3, TIF file.

10.1523/JNEUROSCI.1254-24.2024.f6-4 Figure 6-4 Uncropped immunoblot images for Figure 6f. Download Figure 6-4, TIF file.

### Mitochondria of BACHD-SD mice expressing phosphomimetic full-length mHTT contain less mHTT

As shown, N17 phosphomimetic substitution of mHTT results in reduced mitochondrial localization, TIM23 binding, perturbation of mitochondrial functions, and cell toxicity. Thus, there may be a correlation between the amount of mHTT targeted to the mitochondria and the severity of HD pathology. Indeed, unlike BACHD mice expressing full-length mHTT which develop HD-like pathology, transgenic BACHD-SD mice expressing full-length mHTT with phosphomimetic mutations in N17 terminus (S13D S16D) completely lack a pathological phenotype (Gu et al., 2009). We evaluated whether the attenuated phenotype of these mice could, in part, be the result of decreased mitochondrial localization of mHTT. We pooled two sets, each containing five brains of BACHD and BACHD-SD mice, to isolate mitochondrial fractions to run two independent biological repeats of immunoprobing for mHTT protein content in brain mitochondria. Although we cannot statistically demonstrate a quantifiable difference since only two biological replicates are possible, our data suggest that mitochondria isolated from BACHD-SD mice contain less full-length phosphomimetic mHTT compared with BACHD expressing full-length HTT with no phosphomimetic mutations in the N-terminal sequence (Fig. 6*D*,*E*; Extended Data Fig. 6-3), correlating the reduced presence of mHTT in mitochondria with the benign phenotype in BACHD-SD mHTT-expressing mice. Although we were interested in performing additional experiments with these mice, they are no longer available.

### Phosphorylated endogenous mHTT is less likely to be found inside mitochondria

To confirm that our phosphomimetic constructs model endogenous HTT localization, we compared whole brain lysate with isolated mitochondria from zQ175KI mice and immunoblotted with an anti-HTT antibody that is specific to HTT phosphorylated at the S13 residue (Cariulo et al., 2020) and then reprobed for total HTT. Because HTT is more abundant in cytosol than mitochondria, three times more mitochondrial protein was loaded as compared with whole brain lysate to enable better quantification. To control for loading and different antibody affinity during the phospho-HTT analysis, we compared the ratio of phospho-HTT to total HTT in each sample. If phospho-mHTT is preferentially excluded from the mitochondria, then the ratio of phospho-mHTT to total mHTT should be lower in the mitochondria than in the cytoplasm. Indeed, our data, with values expressed relative to wtHTT found in whole brain lysate of wild-type mice, show that the ratio of phospho-mHTT to total mHTT is lower in mitochondria than whole brain lysates (Fig. 6*F*,*G*). We note that although phospho-wtHTT to total wtHTT also appeared consistently lower in mitochondria than in whole brain lysate (Fig. 6*F*,*G*; Extended Data Fig. 6-4), this difference was only statistically valid for mHTT.

Taken together, these data point to the etiology of the milder pathology in phosphomimetic mice, at least in part, resulting from reduced mitochondrial targeting and diminished association of the mHTT phosphomimetic with TIM23 in mitochondria.

## Discussion

mHTT-mediated pathology is strongly associated with mitochondrial dysfunction (Ratovitski et al., 2012; Jin et al., 2016). HTT does not have a canonical mitochondrial targeting sequence, and one group does not find HTT in the mitochondria (Hamilton et al., 2020). Nonetheless, multiple other reports have demonstrated that fragments and full-length mHTT localize to and associate with mitochondria (Panov et al., 2002; Yu et al., 2003; Choo et al., 2004; Orr et al., 2008; Yano et al., 2014; Yablonska et al., 2019). Therefore, in this study, we presented high-resolution imaging data performed on live cells and fixed enlarged mitochondria to conclusively demonstrate the distribution of HTT inside the mitochondria and reconfirm results published by our, and other independent, laboratories.

We next focused on the mechanism controlling mitochondrial localization. Posttranslational modification controls the charge and structure of proteins, which thereby influences protein–protein interactions as well as intracellular localization. HTT is a primarily cytosolic protein containing numerous phosphorylation and acetylation sites arranged in clusters along its amino acid chain (Ratovitski et al., 2017). Site-directed modifications of mHTT phosphorylation sites alter neuronal toxicity and mitochondrial morphology and membrane potential (Arbez et al., 2017). The remarkably benign phenotype of BACHD-SD mice harboring two phosphomimetic mutations at the N terminus of mHTT suggests that posttranslational modifications of this sequence are essential for mHTT-mediated toxicity (Gu et al., 2009).

Additional studies have suggested a potential role for the HTT N-terminal regulatory domain in the pathogenesis of HD because N17 of HTT impacts its subcellular localization (Atwal et al., 2007; Rockabrand et al., 2007; Maiuri et al., 2013) and degradation (Thompson et al., 2009). The N17 sequence of HTT forms an amphipathic α-helix that enables HTT association with membranes (Michalek et al., 2013; Groover et al., 2020), and we hypothesize that this facilitates HTT's translocation through the mitochondrial protein import complex.

The N17-eGFP construct was designed to evaluate the ability of the first 17 aa to facilitate mitochondrial import, and using multiple complementary experiments, we demonstrate that it has mitochondrial targeting properties. Using microscopy to visualize TMRM-identified mitochondria and N17-eGFP–labeled constructs, we found eGFP fluorescence in mitochondria indicating mitochondrial targeting of eGFP by N17. Using complementary assays (immunoblot assays on isolated mitochondria treated with trypsin; expansion microscopy), we demonstrate N17-eGFP localization inside the mitochondria. We found that N17 phosphomimetic mutations (S13ES16E) result in a significant reduction of mitochondrial localization of both N17 and HTT fragments. Furthermore, we and others demonstrate that phosphomimetic mutations of N17 that disrupt the helical conformation lead to increased nuclear translocation of HTT (Atwal et al., 2011). These data provide further evidence that the N17 domain is a “bona fide” noncanonical mitochondrial targeting sequence for HTT that is regulated through posttranslational phosphorylation.

Further investigation of N17 targeting properties has shown that only a fraction of longer fragments of HTT (171 aa long) are imported into mitochondria, while the rest remain in the cytosol. This is expected, since HTT fulfills numerous cellular functions in the cytoplasm (Zuccato et al., 2010). The content of phosphomimetic wtHTT and mHTT 171-aa-long fragments was significantly lower in mitochondria and higher in the nucleus than in nonmutated versions, confirming the functional role of unphosphorylated N17 as a mitochondrial targeting sequence.

We previously demonstrated that mitochondria from mice and cell lines expressing mHTT exhibit reduced protein import (Yano et al., 2014) and demonstrated a direct interaction of mHTT with the TIM23 subunit of the inner mitochondrial membrane protein import complex, impacting mitochondrial proteome (Yablonska et al., 2019). Reduced mHTT phosphomimetic levels in mitochondria, in combination with lower TIM23 binding, result in an additive protective effect on mitochondria, mitigating mitochondrial pathology of mHTT. Indeed, phosphomimetic mutations in mHTT rescued, in part, the protein import defect in vitro. mHTT fragments harboring two phosphomimetic mutations (S13E S16E) result in less cell death in a striatal cell line ST-Hdh-Q7/Q7 transfected with mHTT fragments and in rat medium spiny neurons (S13D S16D; Thompson et al., 2009). The same phosphomimetic mutations in full-length mHTT (S13D S16D) result in a significant reduction of mHTT content in mitochondria of BACHD-SD mice and are likely one of the reasons for the remarkably ameliorated HD phenotype in these mice (Gu et al., 2009). Furthermore, the reduction in phosphorylated mHTT content in mitochondria is also demonstrated using a phospho-specific HTT antibody in zQ175KI mouse brain (a knock-in model of HD), confirming that this phenomenon is not an artifact of transgenic expression.

Therefore, we demonstrate that HTT fragments carrying two phosphomimetic mutations (S13E S16E) within the N17 domain show reduction of mitochondrial targeting in vitro and weaker binding to TIM23 protein importing complex that at least partially rescued the mHTT inhibitory effect on protein import and cellular toxicity. Our findings outline a mitochondrial mechanism behind reduced pathology in the murine model expressing full-length phosphomimetic mHTT and emphasize the crucial role of mitochondria in HD.

Reduced mitochondrial toxicity of phosphomimetic mHTT highlights the importance of N17 phosphorylation as leverage to manipulate the neurotoxicity of mHTT in HD by regulating cellular kinases for therapeutic purposes. In recent studies, two different kinases phosphorylate HTT, lowering mHTT toxicity. The kinase IKKß was responsible for phosphorylating serine 13 and regulated HTT abundance in R6/1 mice and nontransgenic controls. It did so by activating autophagy genes, thus linking HTT's autophagy scaffold functions and the activation of autophagy (Ochaba et al., 2019). TBK1 kinase efficiently phosphorylated both N-terminal mHTT fragments and full-length HTT, reducing aggregation and cytotoxicity of mHTT exon 1 fragments. TBK1 effects were due to phosphorylation-dependent activation of autophagy and clearance (Hegde et al., 2020).

### Conclusion

Utilizing super-resolution live imaging and expansion microscopy of isolated mitochondria, and immunoprecipitation of full-length mHTT from human cells, we demonstrated intramitochondrial localization of HTT and interaction with the mitochondrial protein importing complex. Phosphomimetic mutations on the N17 domain of mHTT reduce its binding affinity for the TIM23 subunit, preventing mHTT mitochondrial accumulation, and lessen its inhibitory effect on mitochondrial protein import. Our study analyzes the pathway leading to the ameliorated phenotype of BACHD-SD mice and underscores the critical importance of future studies that identify enzymes responsible for regulating HTT phosphorylation and their potential as therapeutic HD targets. We establish a novel key molecular mechanism of mHTT neurotoxicity and suggest phosphorylation of mHTT as a novel target to treat HD neurodegeneration.

## Data Availability

The datasets used and/or analyzed during the current study are available from the corresponding author upon reasonable request.
