# Supplementary figures and images for: Regulation of Mutant Huntingtin Mitochondrial Toxicity by Phosphomimetic Mutations within Its N-Terminal Region
Source: J Neurosci. 2025 Jan 8;45(8):e1254242024. doi: 10.1523/JNEUROSCI.1254-24.2024 (PMC11841767; doi:10.1523/JNEUROSCI.1254-24.2024)

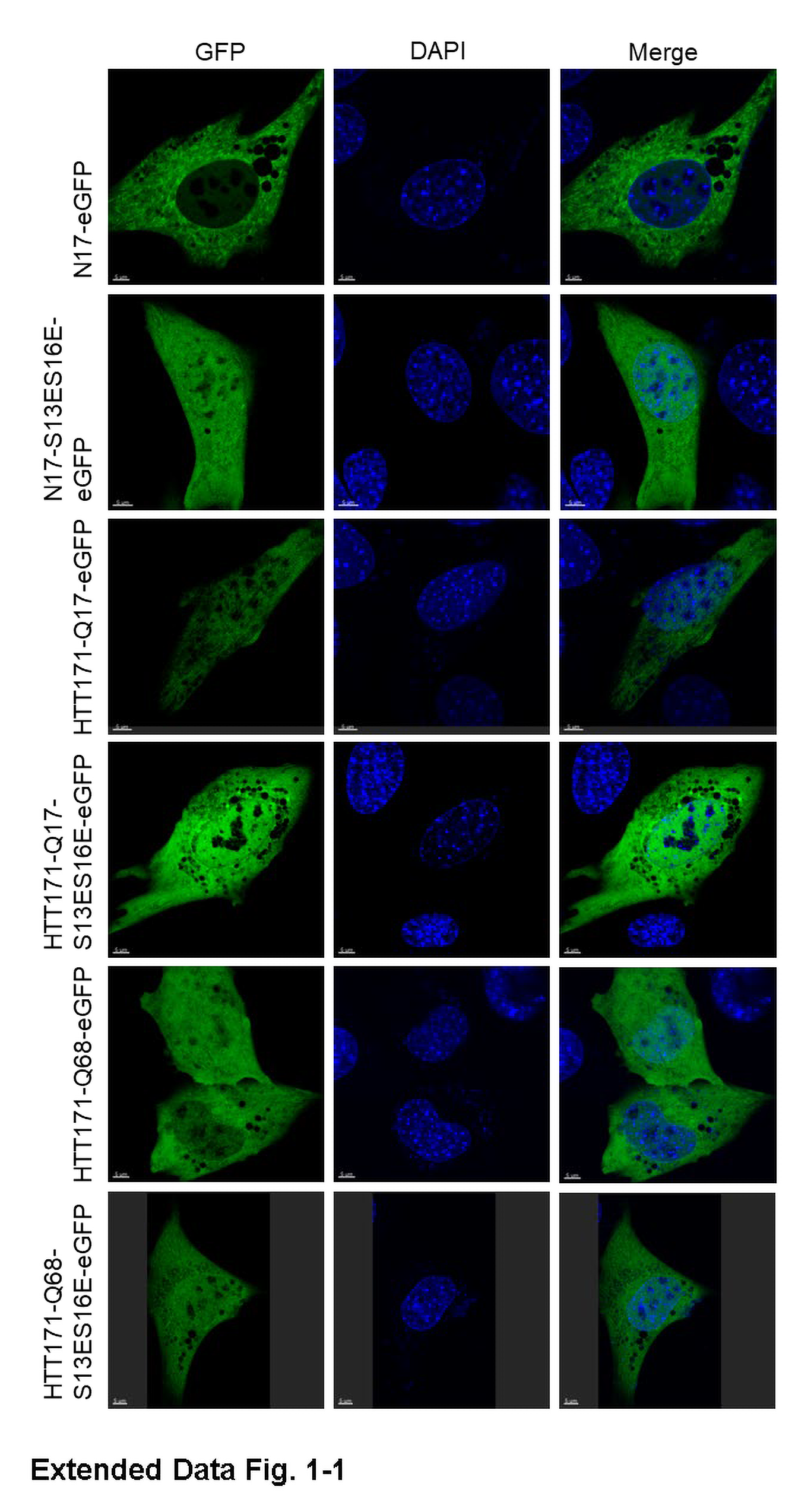

Supplement: Figure 1-1 — Nuclear staining (DAPI-blue) of ST-Hdh-Q7/Q7 cells expressing phosphomimetic (S13ES16E) and non-mutated counterparts of N17-eGFP, HTT171-Q17-eGFP and -Q68-eGFP constructs. Representative images of super resolution microscopy showing distribution of eGFP signal. Download Figure 1-1, TIF file. [file jneuro-45-e1254242024-s001.tif]

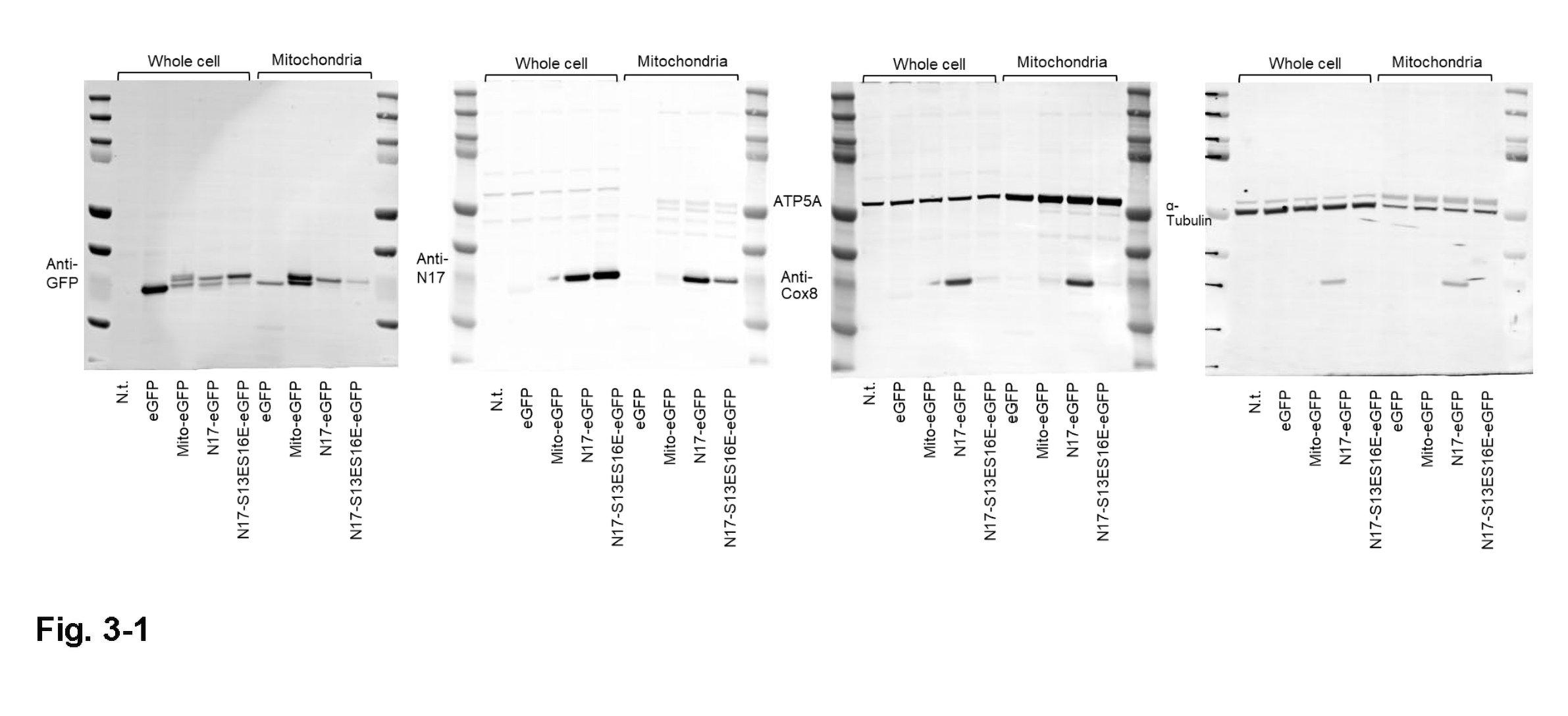

Supplement: Figure 3-1 — Uncropped immunoblot images for Figure 3a. Download Figure 3-1, TIF file. [file jneuro-45-e1254242024-s002.tif]

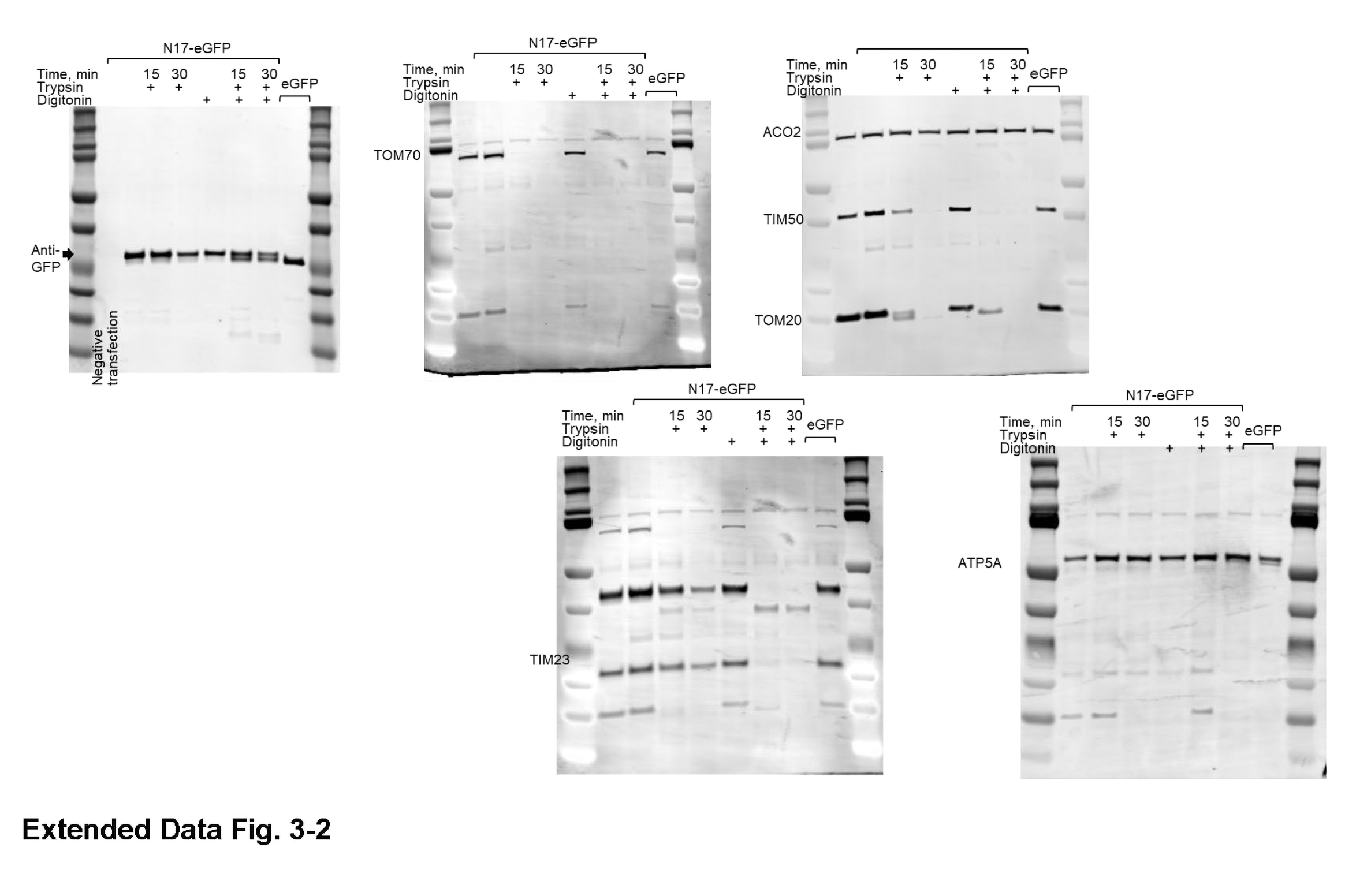

Supplement: Figure 3-2 — Uncropped immunoblot images for Figure 3d. Download Figure 3-2, TIF file. [file jneuro-45-e1254242024-s003.tif]

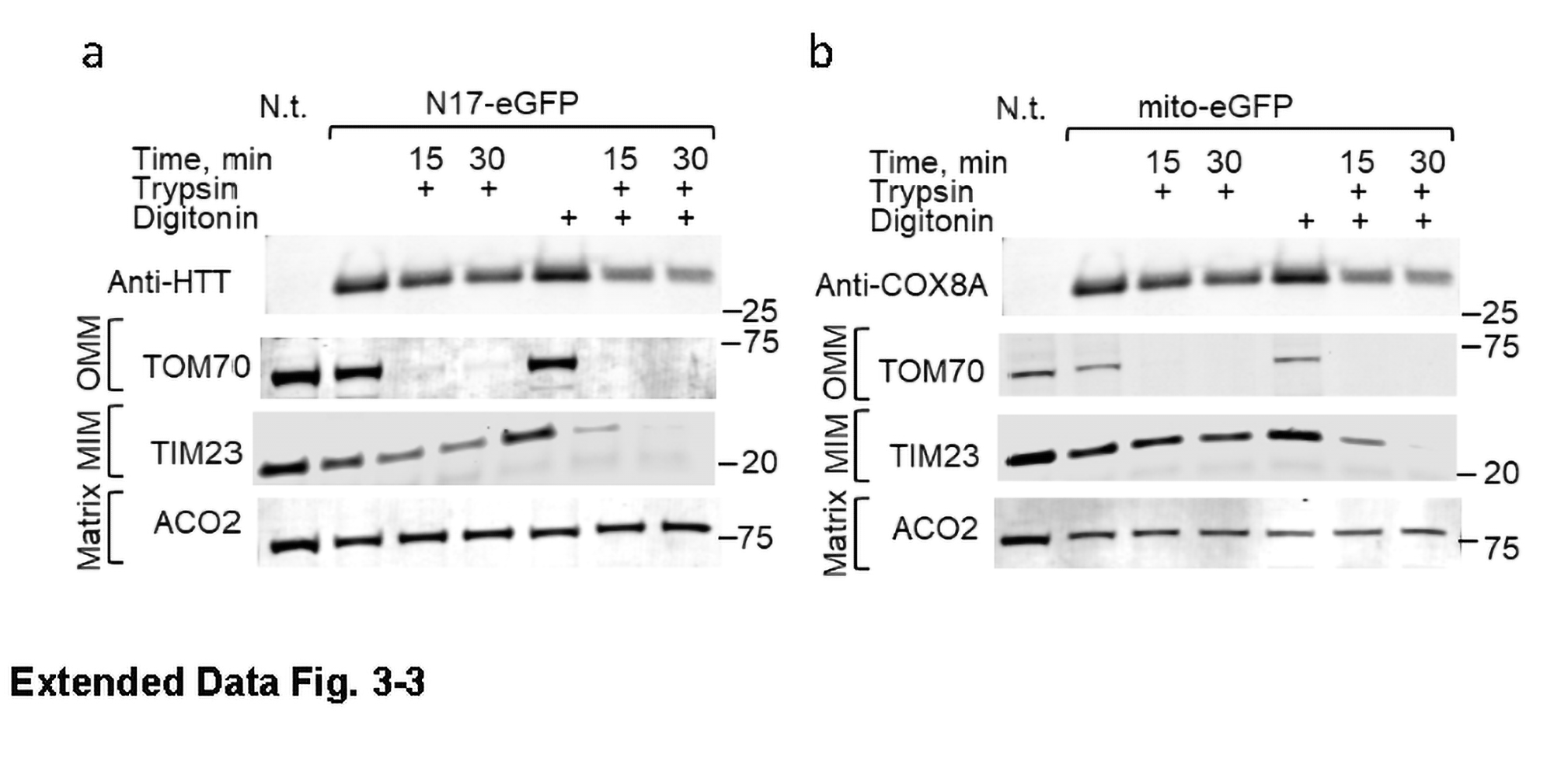

Supplement: Figure 3-3 — N17-terminal amino acid sequence of HTT and mitochondrial targeting sequence of COX8A induce eGFP mitochondrial translocation. (a) Representative immunoblot showing digestion pattern of N17-eGFP (a) and mito-eGFP (b) in mitochondria of HEK293t cells. Mito-eGFP serves as control for intra-mitochondrial/matrix localization due to the canonical COX8A mitochondrial targeting signal. The expression of proteins was detected with anti-HTT antibody that recognizes N-terminal sequence of HTT (a), and anti-COX8A antibody (b). After stripping blots were probed for markers for mitochondrial sub-compartment comparisons: TOM70 for outer mitochondrial membrane (OMM), TIM23 for mitochondrial inner membrane (MIM), and ACO2 for matrix. Cells were harvested for mitochondria isolation 48 hours post-transfection, N.t. – negative transfection sample. Download Figure 3-3, TIF file. [file jneuro-45-e1254242024-s004.tif]

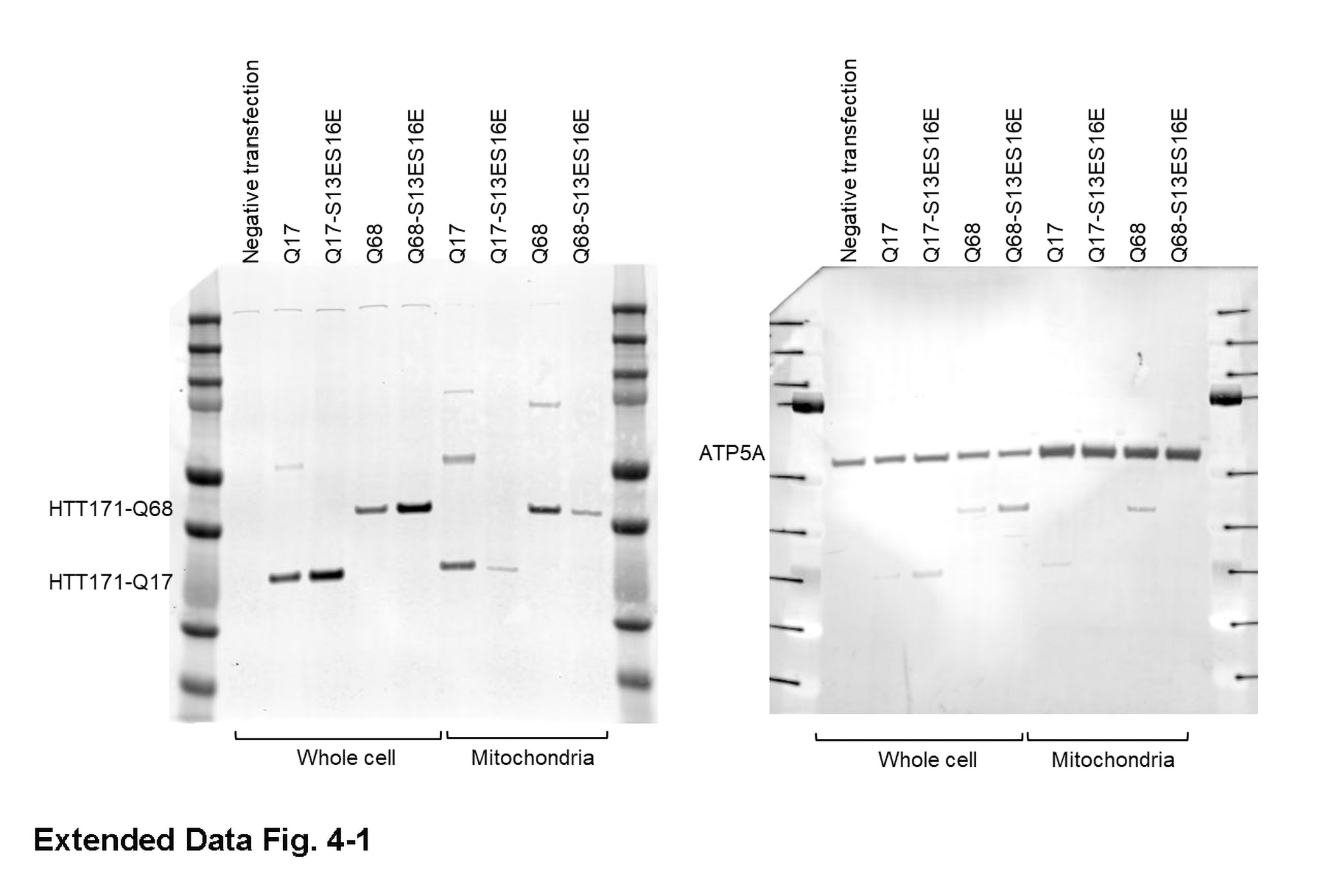

Supplement: Figure 4-1 — Uncropped immunoblot images for Figure 4a. Download Figure 4-1, TIF file. [file jneuro-45-e1254242024-s005.tif]

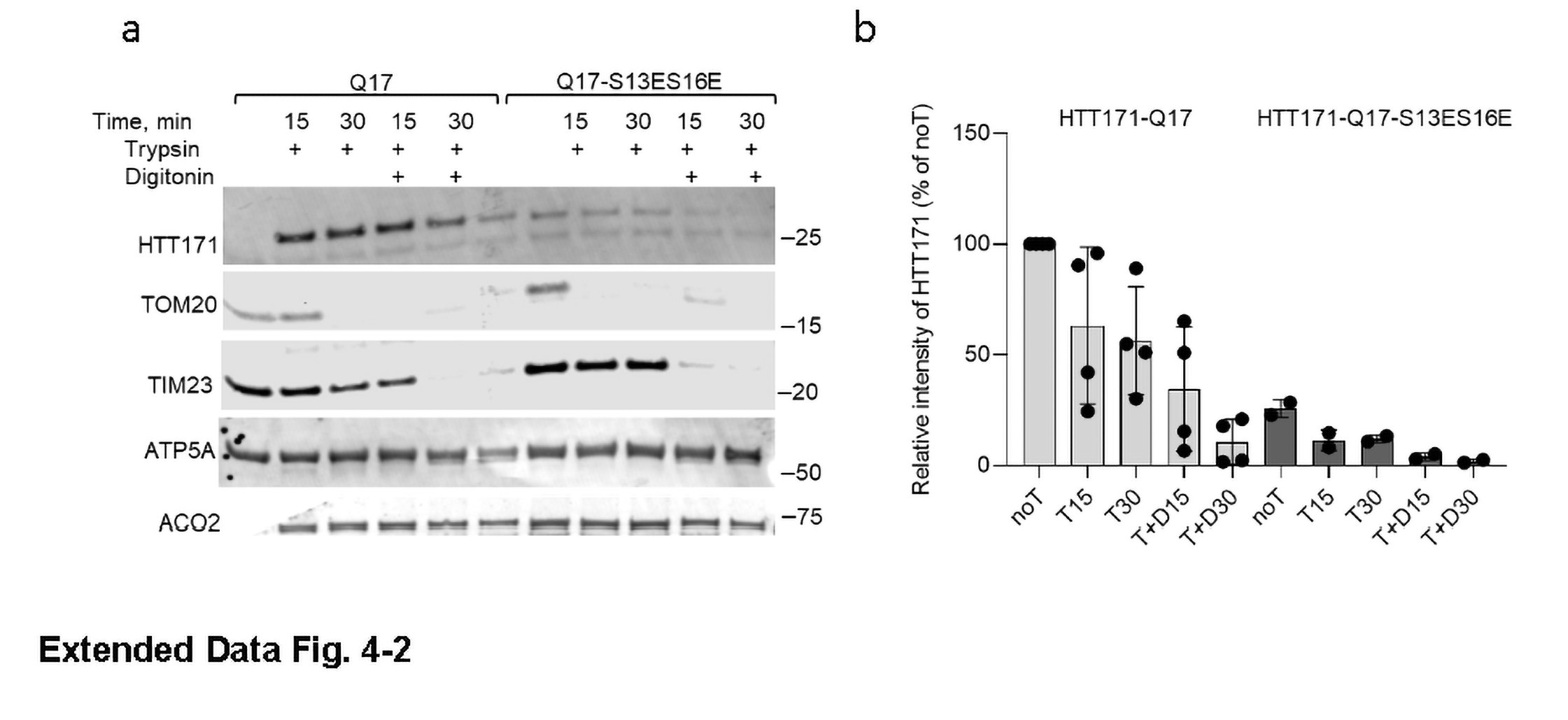

Supplement: Figure 4-2 — The phosphomimetic mutations reduce the amount of wtHTT fragment in mitochondria. Representative immunoblot (a) and quantification (b) demonstrating localization in mitochondria of wtHTT phosphomimetic (HTT171-Q17-S13ES16E) comparing to non-mutated counterpart (HTT171-Q17). Expression of both proteins in HEK293t mitochondria was detected with anti-flag antibody. After stripping blots were probed for markers for mitochondrial sub-compartment comparisons: TOM20 for OMM, TIM23 for MIM, and ATP5A, ACO2 for matrix. Immunoblot bands were normalized to the untreated sample (noT stands for no trypsin), which was taken as 100%. Download Figure 4-2, TIF file. [file jneuro-45-e1254242024-s006.tif]

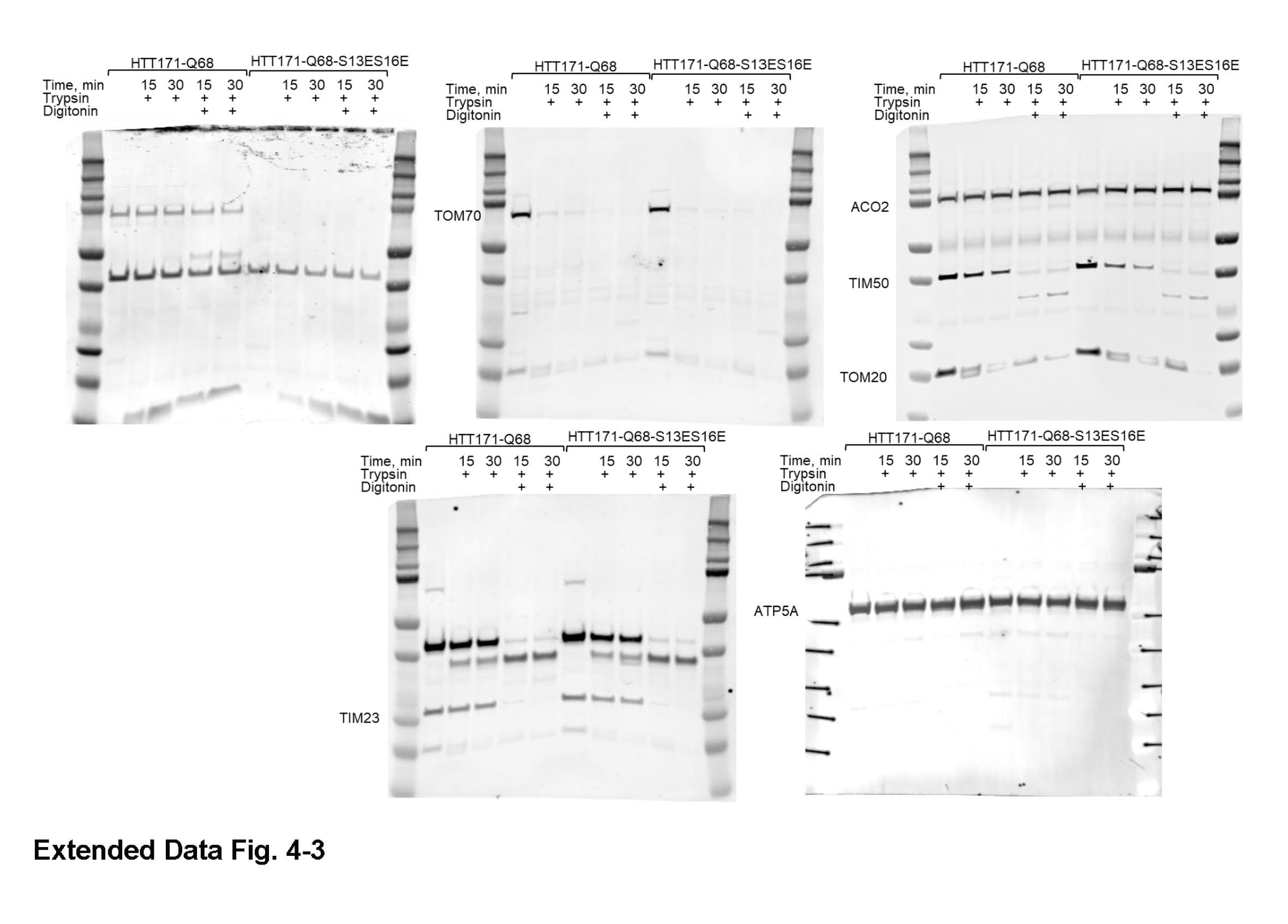

Supplement: Figure 4-3 — Uncropped immunoblot images for Figure 4d. Download Figure 4-3, TIF file. [file jneuro-45-e1254242024-s007.tif]

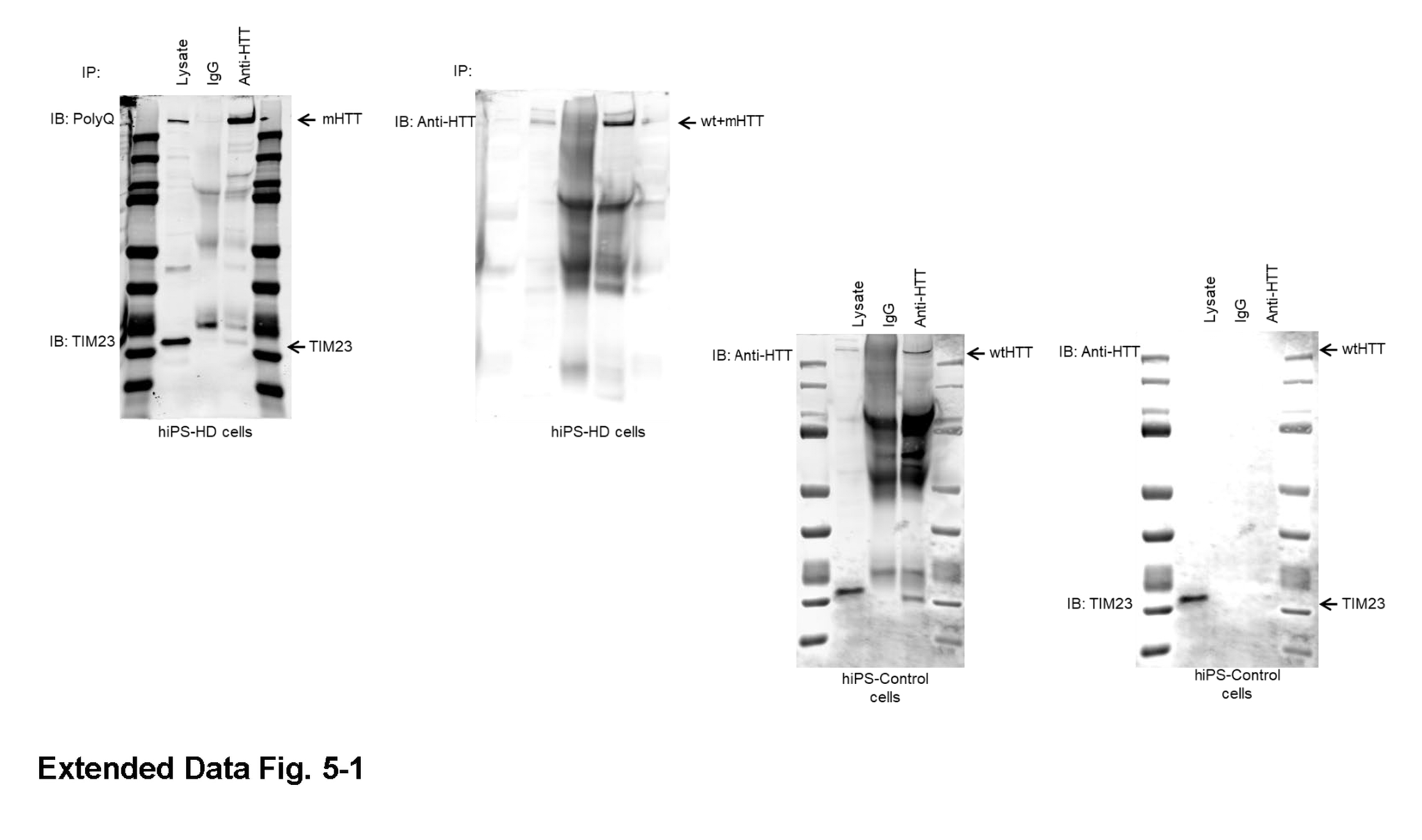

Supplement: Figure 5-1 — Uncropped immunoblot images for Figure 5a. Download Figure 5-1, TIF file. [file jneuro-45-e1254242024-s008.tif]

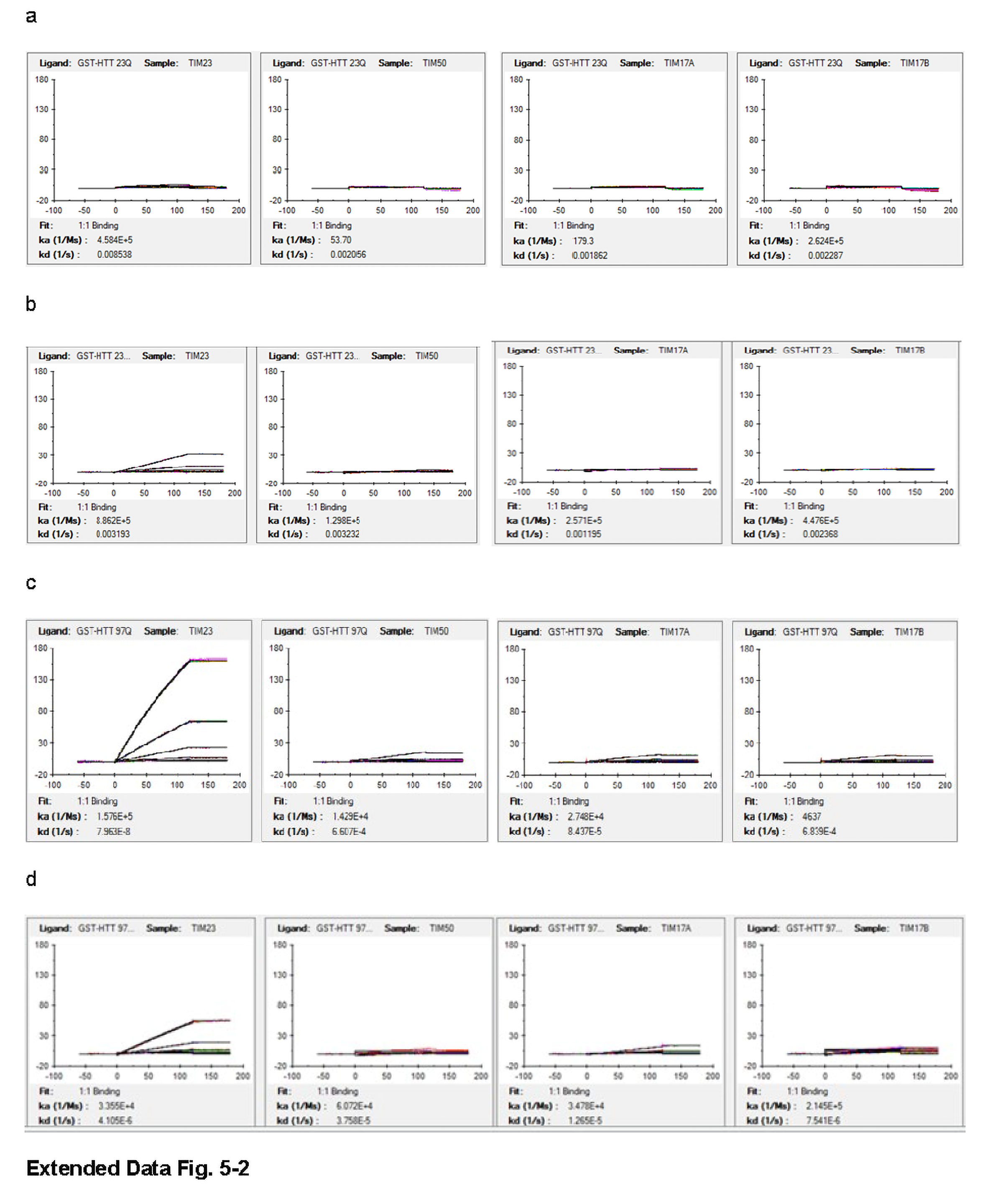

Supplement: Figure 5-2 — Representative SPR sensorgrams displaying binding of TIM23, TIM50, TIM17A and TIM17B proteins (sample) to immobilized ligand wild type HTT (HTTex1-23Q) (a), wild type HTT phosphomimetic (GST-HTTex1-23Q-S13ES16E) (b), mutant HTT (GST-HTT-97Q) (c) and mutant HTT phosphomimetic (GST-HTT-97Q-S13ES16E) (d). Download Figure 5-2, TIF file. [file jneuro-45-e1254242024-s009.tif]

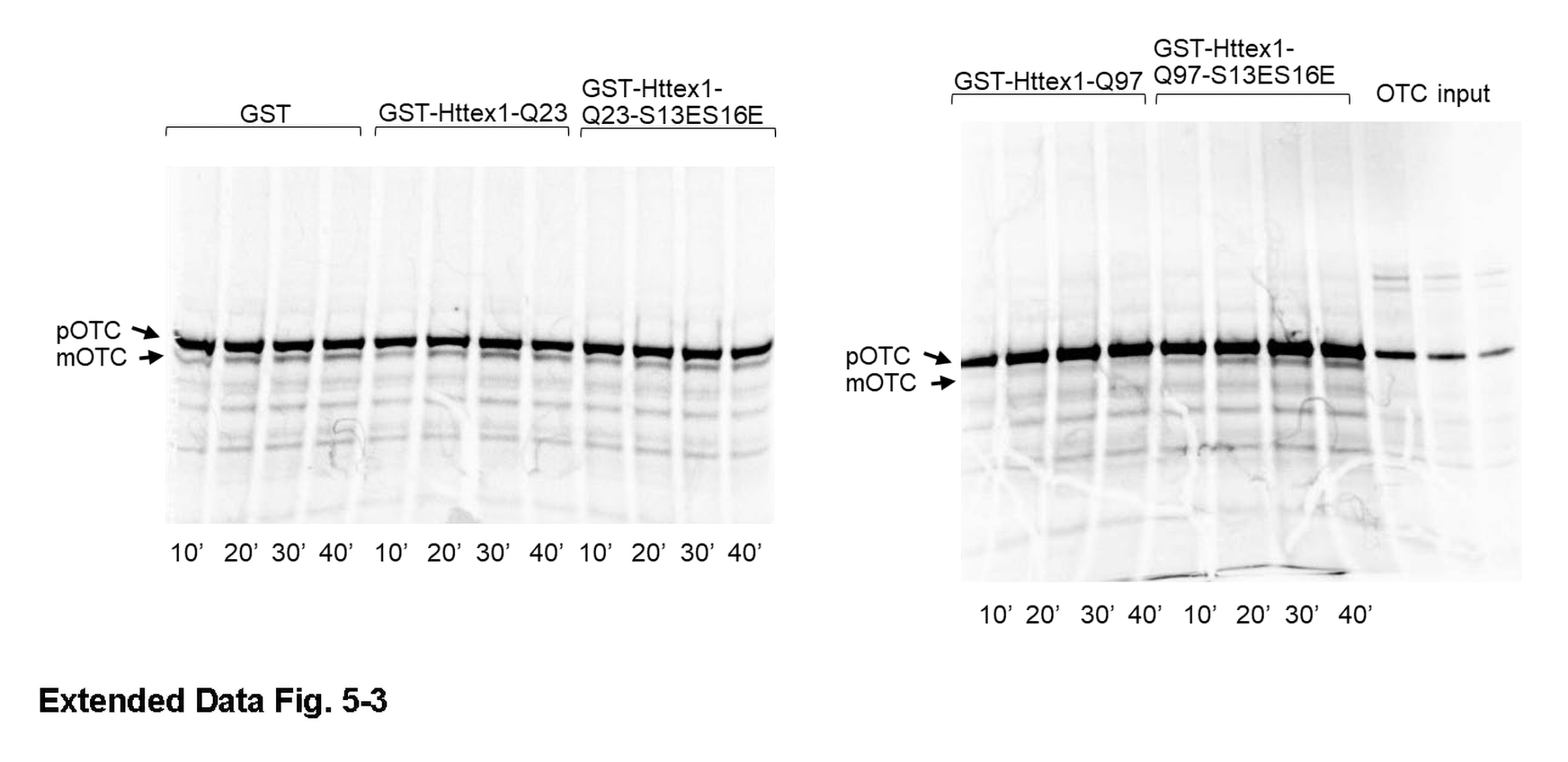

Supplement: Figure 5-3 — Uncropped immunoblot images for Figure 5c. Download Figure 5-3, TIF file. [file jneuro-45-e1254242024-s010.tif]

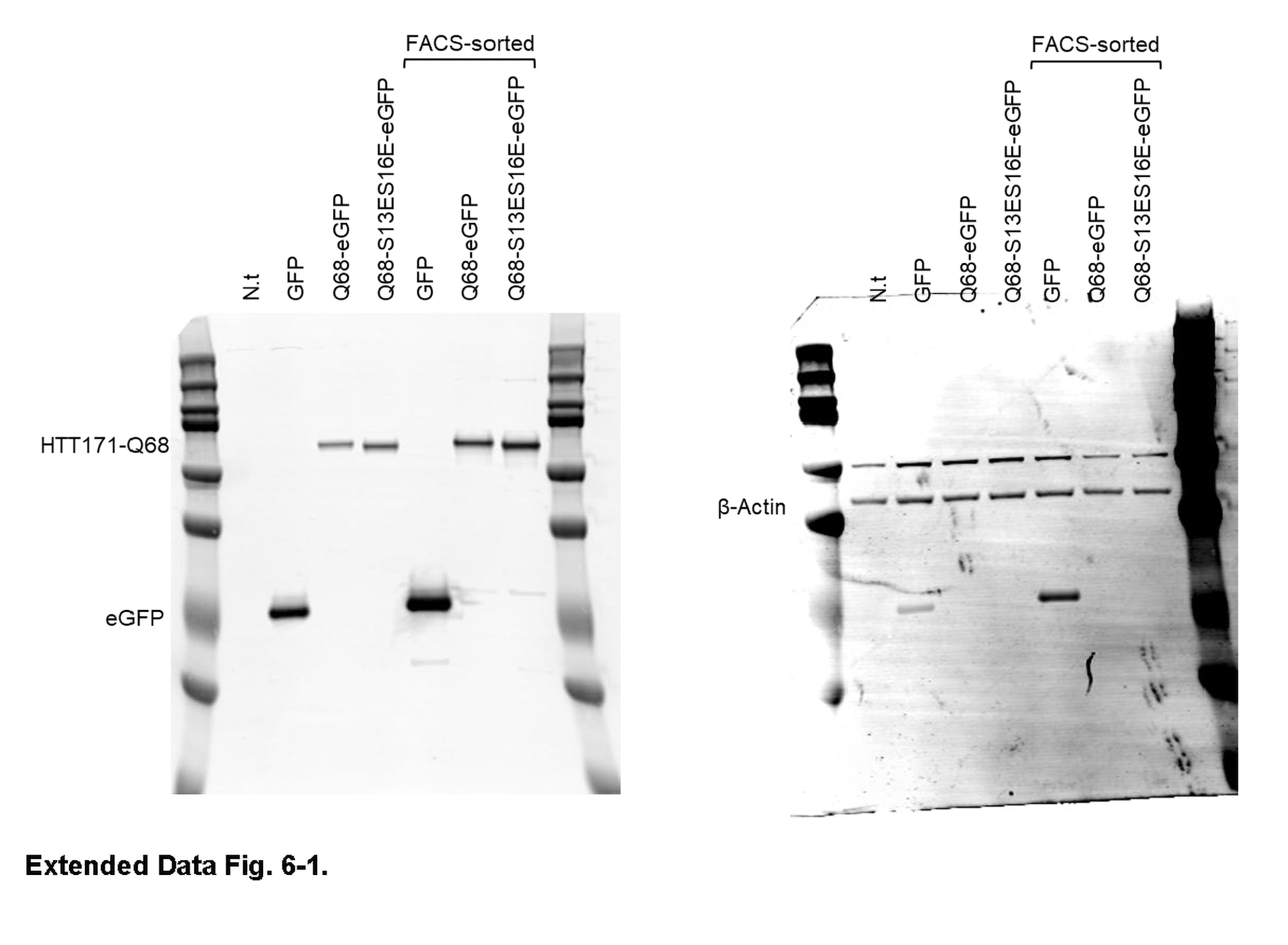

Supplement: Figure 6-1 — Uncropped immunoblot images for Figure 6a. Download Figure 6-1, TIF file. [file jneuro-45-e1254242024-s011.tif]

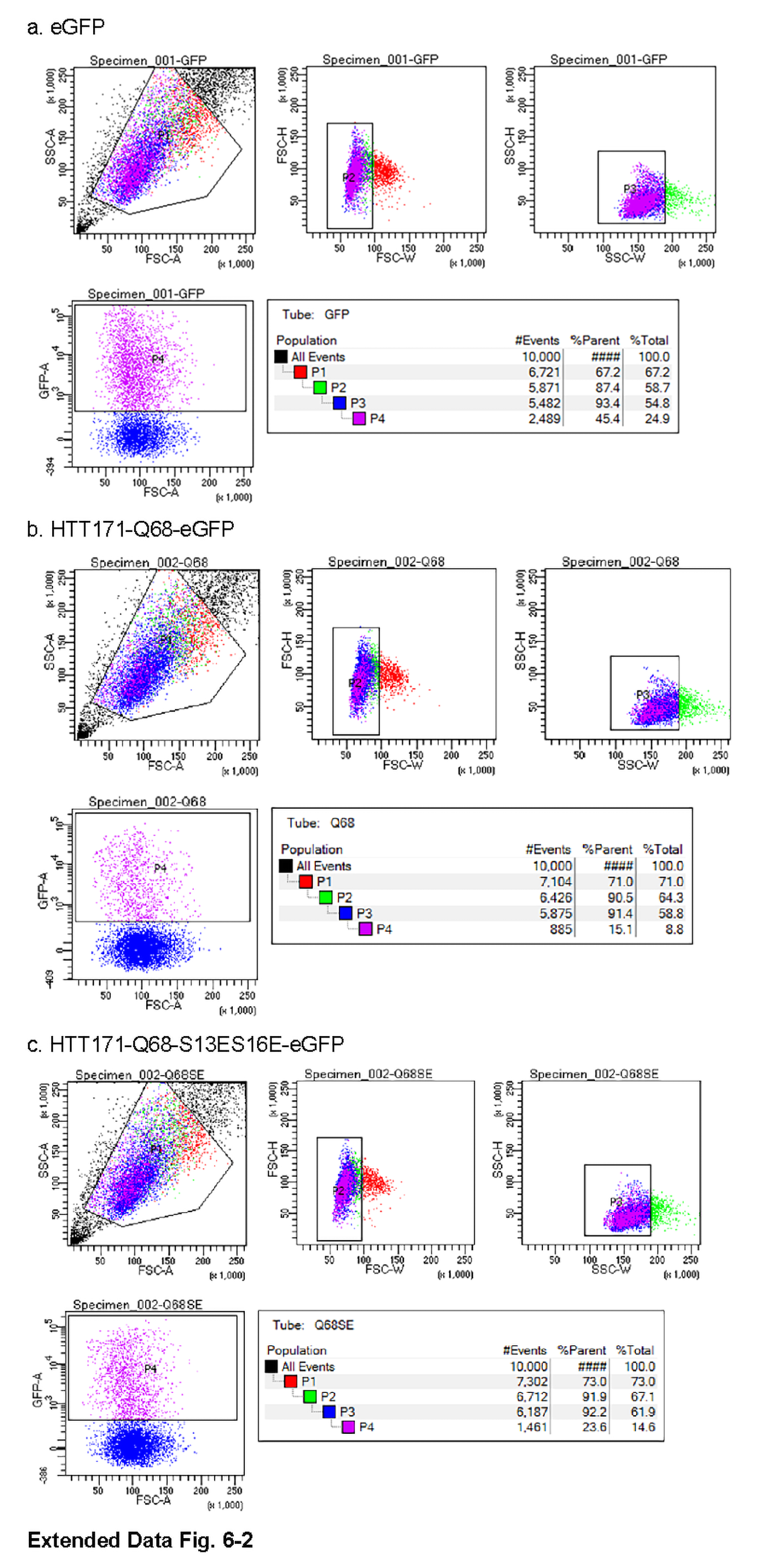

Supplement: Figure 6-2 — Flow cytometry charts of eGFP positive populations of ST-Hdh-Q7/Q7 cells collected 48 h post-transfection expressing eGFP (a), HTT171-Q68 (b) or HTT171-Q68-S13ES16E-eGFP (c). Cells were re-plated immediately into 96-well plates. Download Figure 6-2, TIF file. [file jneuro-45-e1254242024-s012.tif]

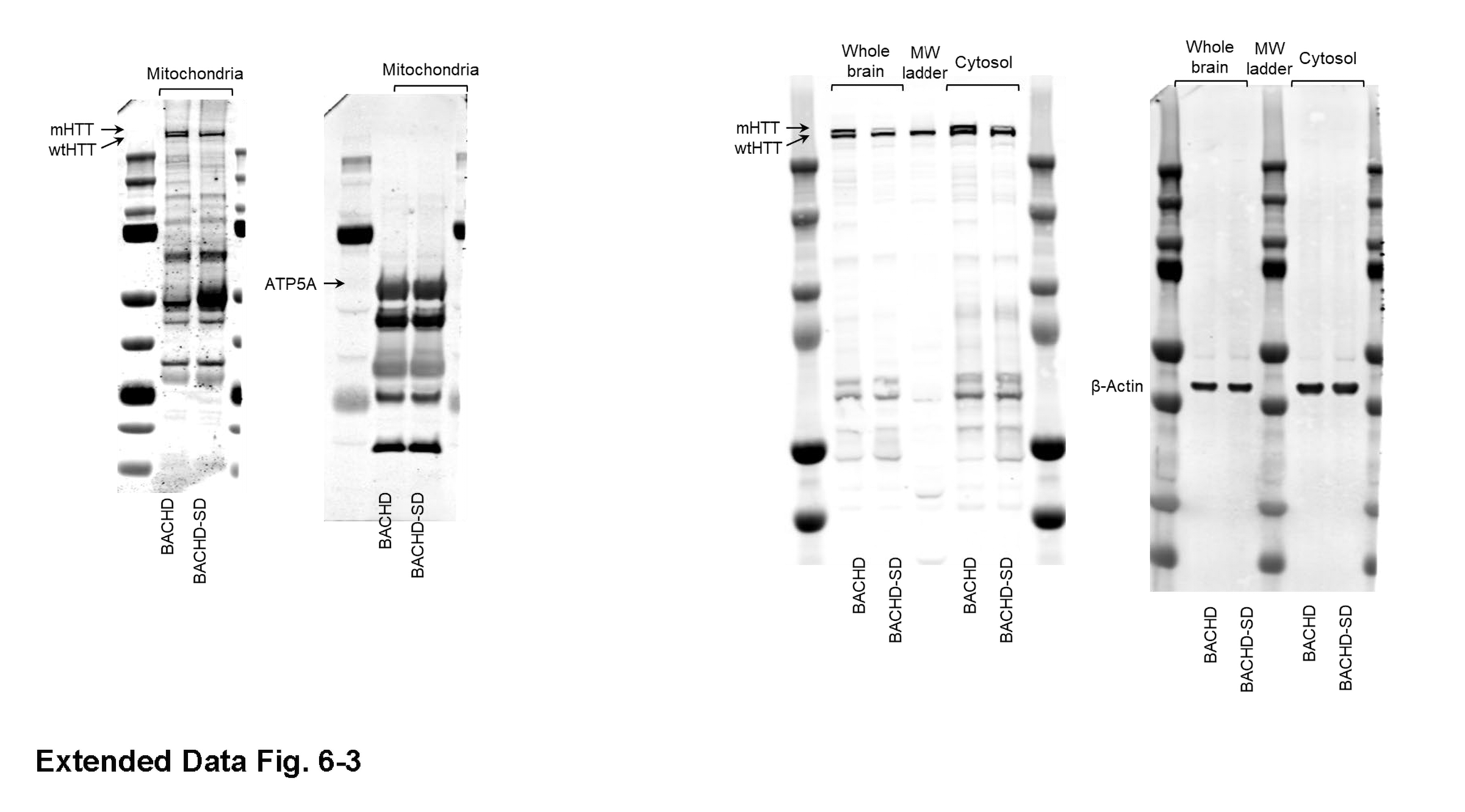

Supplement: Figure 6-3 — Uncropped immunoblot images for Figure 6d. Download Figure 6-3, TIF file. [file jneuro-45-e1254242024-s013.tif]

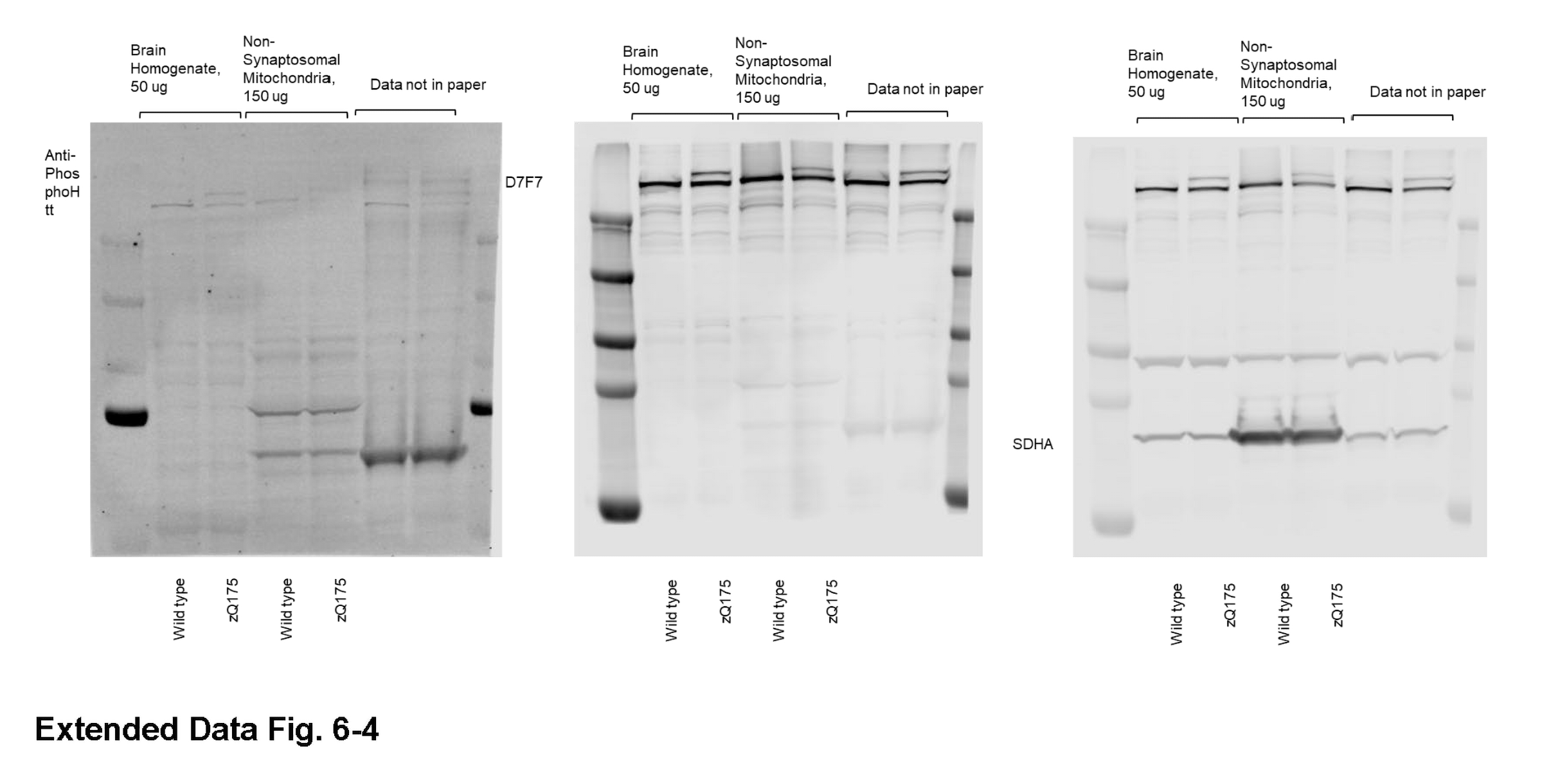

Supplement: Figure 6-4 — Uncropped immunoblot images for Figure 6f. Download Figure 6-4, TIF file. [file jneuro-45-e1254242024-s014.tif]
