# Supplementary material for: Regulation of Mutant Huntingtin Mitochondrial Toxicity by Phosphomimetic Mutations within Its N-Terminal Region
Source: J Neurosci. 2025 Jan 8;45(8):e1254242024. doi: 10.1523/JNEUROSCI.1254-24.2024 (PMC11841767; doi:10.1523/JNEUROSCI.1254-24.2024)
Supplement: Table 5-1 — Binding affinity constants of TIM23 complex subunits with mHTT exon1. Download Table 5-1, DOCX file. [file jneuro-45-e1254242024-s015.docx]

**Extended Data Table 5-1** Binding affinity constants of TIM23 complex subunits with mHTT exon1.

| Ligand | Sample | Curve | Temp (°C) | Fit | k_a_ | k_d_ | KD |
| --- | --- | --- | --- | --- | --- | --- | --- |
| GST-HTTex1-23Q | TIM23 | Fc=2-1 | 25 | 1:1 Binding | 1.22E+04 | 4.18E-05 | 3.43E-09 |
| GST-HTTex1-23Q | TIM50 | Fc=2-1 | 25 | 1:1 Binding | 3.87E+05 | 0.2054 | 5.31E-07 |
| GST-HTTex1-23Q | TIM17A | Fc=2-1 | 25 | 1:1 Binding | 2540 | 0.03182 | 1.25E-05 |
| GST-HTTex1-23Q | TIM17B | Fc=2-1 | 25 | 1:1 Binding | 5.38E+05 | 0.0539 | 1.00E-07 |
| GST-HTTex1-23Q-S13ES16E | TIM23 | Fc=2-1 | 25 | 1:1 Binding | 8.86E+05 | 0.003193 | 3.60E-09 |
| GST-HTTex1-23Q-S13ES16E | TIM50 | Fc=2-1 | 25 | 1:1 Binding | 1.30E+05 | 0.003232 | 2.49E-08 |
| GST-HTTex1-23Q-S13ES16E | TIM17A | Fc=2-1 | 25 | 1:1 Binding | 2.57E+05 | 0.001195 | 4.65E-09 |
| GST-HTTex1-23Q-S13ES16E | TIM17B | Fc=2-1 | 25 | 1:1 Binding | 4.48E+05 | 0.002368 | 5.29E-09 |
| GST-HTTex1-97Q | TIM23 | Fc=4-3 | 25 | 1:1 Binding | 1.58E+05 | 7.96E-08 | 5.05E-13 |
| GST-HTTex1-97Q | TIM50 | Fc=4-3 | 25 | 1:1 Binding | 1.43E+04 | 6.61E-04 | 4.62E-08 |
| GST-HTTex1-97Q | TIM17A | Fc=4-3 | 25 | 1:1 Binding | 2.75E+04 | 8.44E-05 | 3.07E-09 |
| GST-HTTex1-97Q | TIM17B | Fc=4-3 | 25 | 1:1 Binding | 4637 | 6.84E-04 | 1.48E-07 |
| GST-HTTex1-97Q-S13ES16E | TIM23 | Fc=2-1 | 25 | 1:1 Binding | 3.36E+04 | 4.11E-06 | 1.22E-10 |
| GST-HTTex1-97Q-S13ES16E | TIM50 | Fc=2-1 | 25 | 1:1 Binding | 6.07E+04 | 3.76E-05 | 6.19E-10 |
| GST-HTTex1-97Q-S13ES16E | TIM17A | Fc=2-1 | 25 | 1:1 Binding | 3.48E+04 | 1.27E-05 | 3.64E-10 |
| GST-HTTex1-97Q-S13ES16E | TIM17B | Fc=2-1 | 25 | 1:1 Binding | 2.15E+05 | 7.54E-06 | 3.52E-11 |

note:

k_a_ – association constant

k_d_ - dissociation constant

KD - equilibrium dissociation constant
